# Dynamic change of electrostatic field in TMEM16F permeation pathway shifts its ion selectivity

**DOI:** 10.7554/eLife.45187

**Published:** 2019-07-18

**Authors:** Wenlei Ye, Tina W Han, Mu He, Yuh Nung Jan, Lily Yeh Jan

**Affiliations:** 1 Department of PhysiologyUniversity of California, San FranciscoSan FranciscoUnited States; 2 Howard Hughes Medical InstituteSan FranciscoUnited States; 3 Department of Biochemistry and BiophysicsUniversity of California, San FranciscoSan FranciscoUnited States; National Institute of Neurological Disorders and Stroke, National Institutes of HealthUnited States; The University of Texas at AustinUnited States

**Keywords:** TMEM16F, scramblase, ion selectivity, calcium-activated chloride channel, small-conductance cation channel, Mouse

## Abstract

TMEM16F is activated by elevated intracellular Ca^2+^, and functions as a small-conductance ion channel and as a phospholipid scramblase. In contrast to its paralogs, the TMEM16A/B calcium-activated chloride channels, mouse TMEM16F has been reported as a cation-, anion-, or non-selective ion channel, without a definite conclusion. Starting with the Q559K mutant that shows no current rundown and less outward rectification in excised patch, we found that the channel shifted its ion selectivity in response to the change of intracellular Ca^2+^ concentration, with an increased permeability ratio of Cl^-^ to Na^+^ (P_Cl-_/P_Na+_) at a higher Ca^2+^ level. The gradual shift of relative ion permeability did not correlate with the channel activation state. Instead, it was indicative of an alteration of electrostatic field in the permeation pathway. The dynamic change of ion selectivity suggests a charge-screening mechanism for TMEM16F ion conduction, and it provides hints to further studies of TMEM16F physiological functions.

## Introduction

Mammalian TMEM16F (Anoctamin-6, ANO6) is a membrane protein with dual functions of phospholipid scrambling and ion conduction, both activated by elevation of intracellular Ca^2+^ (Whitlock and Hartzell, 2017; Falzone et al., 2018; Bevers and Williamson, 2016; Pedemonte and Galietta, 2014). When activated, TMEM16F mediates the exposure of phosphatidylserine, a lipid normally restricted to the inner leaflet of cell membrane lipid bilayer, to the cell surface (Suzuki et al., 2010). This process, known as lipid scrambling, initiates many physiological processes such as recruitment of tissue factors to the platelet surface for thrombin production to trigger blood coagulation and modulation of immune responses in T lymphocytes (Suzuki et al., 2010; Yang et al., 2012; Hu et al., 2016; Zaitseva et al., 2017). TMEM16F is also a Ca^2+^-activated small-conductance ion channel (Yang et al., 2012), raising the possibility that it has additional physiological functions that have not been revealed. However, there is discrepancy regarding TMEM16F ion selectivity reported by different labs. Recorded with excised-patch inside-out configuration, TMEM16F channels are quickly activated by micromolar Ca^2+^ and they are more permeable to cations (mainly physiological cations such as Na^+^, K^+^ and Ca^2+^) than to Cl^-^ (Yang et al., 2012; Alvadia et al., 2019). Surprisingly, many groups have reported that the TMEM16F whole-cell current is activated several minutes after cytoplasmic Ca^2+^ elevation, and it displays less cation-selectivity (Yu et al., 2015) or even higher permeability to Cl^-^ than to Na^+^ (Grubb et al., 2013; Scudieri et al., 2015; Shimizu et al., 2013; Tian et al., 2012). This disagreement hampers our further understanding of the functions of this membrane protein.

TMEM16F belongs to the mammalian TMEM16 membrane protein family with 10 members (Whitlock and Hartzell, 2017; Falzone et al., 2018; Pedemonte and Galietta, 2014). The founding members TMEM16A (ANO1) and TMEM16B (ANO2) represent the only two canonical Ca^2+^-activated Cl^-^ channels (CaCC) without lipid scrambling functions (Schroeder et al., 2008; Caputo et al., 2008; Yang et al., 2008; Stephan et al., 2009), while several other mammalian homologs show a modest selectivity between cations and anions but with lipid scrambling capacities (Suzuki et al., 2010; Suzuki et al., 2013; Gyobu et al., 2017; Le et al., 2019a; Watanabe et al., 2018), a property closer to that of their fungal homologues (Malvezzi et al., 2013; Brunner et al., 2014; Lee et al., 2016). Mammalian TMEM16 proteins are dimeric, as revealed by structural analyses of TMEM16A, TMEM16F and TMEM16K via electron cryo-microscopy (cryo-EM) or crystallography (Alvadia et al., 2019; Paulino et al., 2017a; Paulino et al., 2017b; Bushell, 2018; Dang et al., 2017; Feng et al., 2019), and each subunit contains a permeation pathway that works independently (Paulino et al., 2017b; Lim et al., 2016; Jeng et al., 2016). For TMEM16A, each subunit contains 10 transmembrane helices (TM1 ~TM10), and the stabilization of TM6 by the binding of Ca^2+^ ions to the binding-pocket formed by acidic residues on TM6, TM7 and TM8 within the transmembrane domain opens the permeation pathway, composed of TM3-TM8 (Paulino et al., 2017a; Peters et al., 2018). Recent studies suggested that, in addition to the pore-lining residues on TM3-TM8 along the TMEM16A permeation pathway for anion conduction, the positive electrostatic field introduced by the Ca^2+^ ions bound to the binding-pocket also contributes to anion accessibility to the pore (Lam and Dutzler, 2018). This finding raised the possibility that TMEM16 proteins might adopt a strategy of utilizing the electrostatic field to control ion accessibility to the pore, a process that allows these proteins to be modulated by their surroundings such as Ca^2+^ level. We hypothesize that TMEM16F shifts its ion selectivity in response to elevation of intracellular Ca^2+^ concentrations. However, because TMEM16F current in inside-out excised patch exhibits rapid desensitization and rundown in high Ca^2+^ (Ye et al., 2018), it is challenging to test for TMEM16F ion permeability ratio under a wide range of Ca^2+^ concentrations.

In TMEM16F, glutamine 559 (Q559) faces the ionic permeation pathway, and lysine substitution of this pore-lining residue (Q559K) reduces the ratio of Na^+^ permeability to Cl^-^ permeability (P_Na+_/P_Cl-_) (Yang et al., 2012; Alvadia et al., 2019; Feng et al., 2019). Previous studies also show that the current of Q559K persists with prolonged exposure of the excised patch to high intracellular Ca^2+^, and that it displays reduced outward rectification (Alvadia et al., 2019; Nguyen et al., 2019). In this study, we found that this mutant has different ratio of Na^+^ permeability to Cl^-^ permeability (P_Na+_/P_Cl-_) in different Ca^2+^ concentrations. The shift of permeability ratio does not correlate with alterations of channel open states, but instead is regulated by the change of electrostatic field along the permeation pathway, on which divalent cations such as Ca^2+^ and Zn^2+^ have more significant impact than monovalent ions. Depolarization, which facilitates intracellular cation entry into the membrane electric field, promotes the shift of the relative permeability toward a preference for Cl^-^ over cations. Such an electrostatic modulation could reflect a general feature of the mechanism of ionic transportation by TMEM16 proteins, and it suggests that TMEM16F harbors an inherent machinery that allows it to dynamically modulate preference between cations and anions in response to its local environment.

## Results

### TMEM16F Q559K increases permeability to Cl^-^ as intracellular Ca^2+^ increases

**Figure 1.**
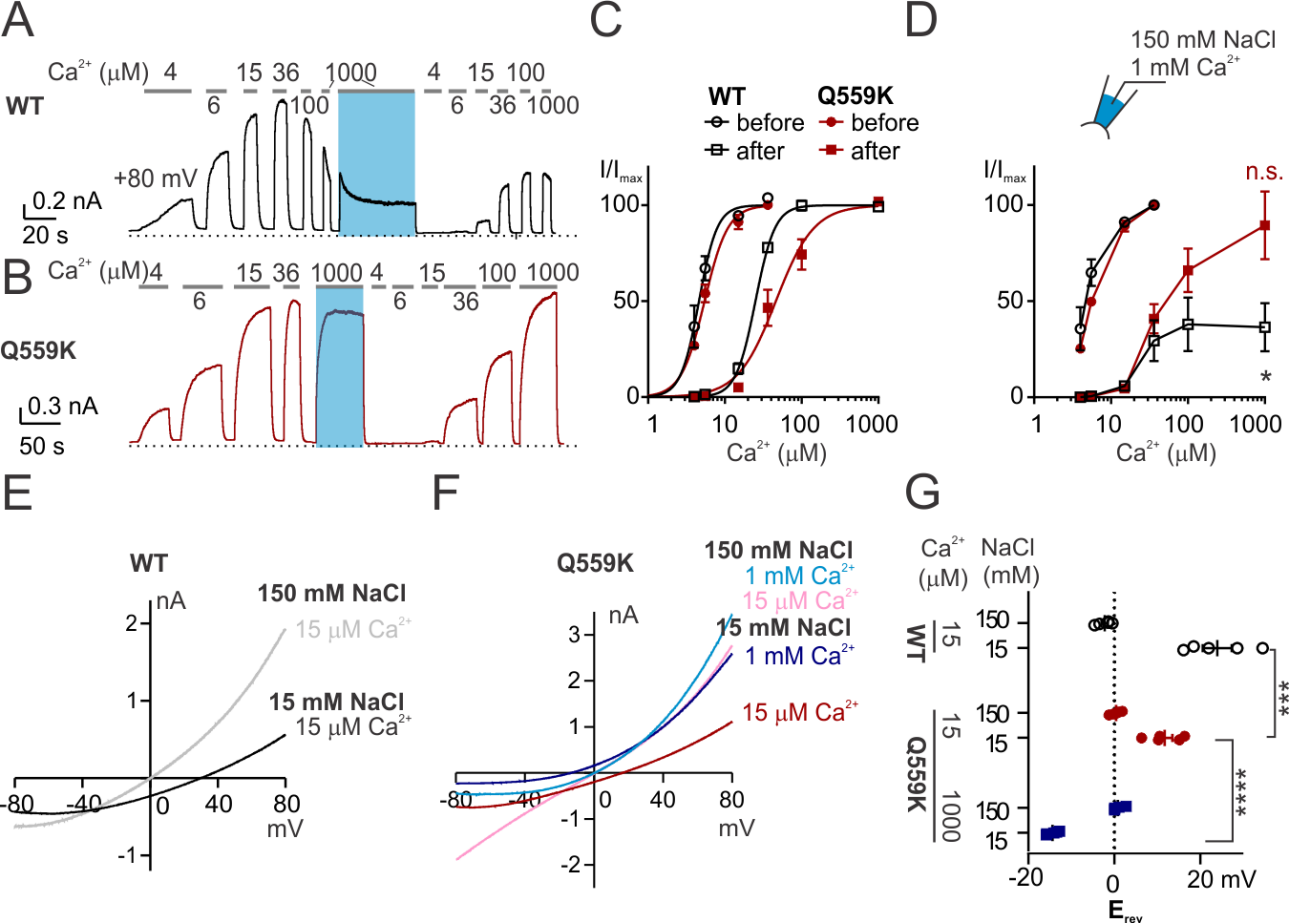
TMEM16F Q559K shifts its reversal potential in response to change of intracellular Ca^2+^ concentration. (**A, B**) Representative recordings of TMEM16F wild type (WT) and Q559K in different Ca^2+^ concentrations. Traces were recorded from transfected HEK293 cells and the inside-out patches were held at +80 mV. The shades illustrate 1 min treatment with 1 mM Ca^2+^ that catalyzes PIP_2_ degradation by membrane-tethered phospholipase. (**C**) Dose-response curves for Ca^2+^-activation of WT and Q559K before and after 1 mM Ca^2+^ treatment, respectively. Currents before and after 1 mM Ca^2+^ were separately fitted to the Hill equation and normalized to their respective maximal amplitudes. (**D**) Change of current magnitudes of WT and Q559K. The currents were normalized to the maximal magnitudes before 1 mM Ca^2+^ for each cell. *p<0.05 (one sample *t* test against hypothetical value ‘1’). (**E, F**) Representative I-V relationships of WT and Q559K recorded in indicated conditions. The traces were recorded with a hyperpolarizing ramp from +80 mV to −80 mV (−1 V/s) following holding at +80 mV. (**G**) Scatter plot of reversal potentials (E_rev_) obtained from traces as in *E* and *F* without background correction. *p*-Values were determined with Sidak's multiple comparisons following two-way ANOVA.

Recording from inside-out membrane patch held at +80 mV revealed that wild-type mouse TMEM16F current was activated by intracellular Ca^2+^ in a dose-dependent manner. The current started to decrease when Ca^2+^ was higher than ~30 µM, as a result of both desensitization and rundown (Figure 1A,C,D) (Ye et al., 2018). Here, desensitization refers to a decreased sensitivity to Ca^2+^, caused by degradation of PIP_2_ via membrane-tethered phospholipase activated by high intracellular Ca^2+^. Desensitization is also reflected by the requirement of stronger depolarization for channel activation, as a result of the synergy between depolarization and Ca^2+^ level (to be shown later). Rundown refers to a reduction of current magnitude (induced by 1 mM Ca^2+^ in this case). To measure the voltage dependence of activation, we recorded with a voltage-family protocol from −40 mV to +160 mV with 10 mV increments followed by holding at 100 mV to obtain ‘tail-currents’, which we used as indicators of steady-state conductance at the range of voltages. Fitting to the sigmoidal conductance-voltage (G-V) relationship yielded V_1/2_ of 76 ± 11 mV in 15 µM Ca^2+^ for wild-type TMEM16F, while in 1 mM Ca^2+^, desensitization strongly reduced the voltage-gating, as was previously reported (Figure 1—figure supplement 1A,B,C) (Ye et al., 2018). The combination of rundown and desensitization rendered it difficult to record the reversal potential (E_rev_) of wild-type TMEM16F in 1 mM Ca^2+^. In our experiments, we measured the E_rev_ from the current recorded with a hyperpolarizing ramp (from +80 mV to −80 mV, 1 V/s) following holding the excised patch at +80 mV. When we switched the bath (equivalent to intracellular solution) from 150 mM NaCl to 45 mM or 15 mM NaCl (osmolarity balanced with mannitol), the current recorded from *Tmem16f*-transfected cells immediately diminished with the hyperpolarizing voltage and was indistinguishable from the background current endogenous to HEK293 cells (Figure 1—figure supplement 2E,F). For TMEM16F current induced by 15 µM Ca^2+^ before desensitization, despite the channel closing with the hyperpolarizing voltage, the remaining current was still large enough, distinguishable from the current recorded in 0 Ca^2+^ (referred to as ‘background current’, Figure 1—figure supplement 2B,C). Consistent with previous reports (Yang et al., 2012; Alvadia et al., 2019), it was moderately selective for Na^+^ over Cl^-^ (Figure 1E).

**Figure 1—figure supplement 1.**
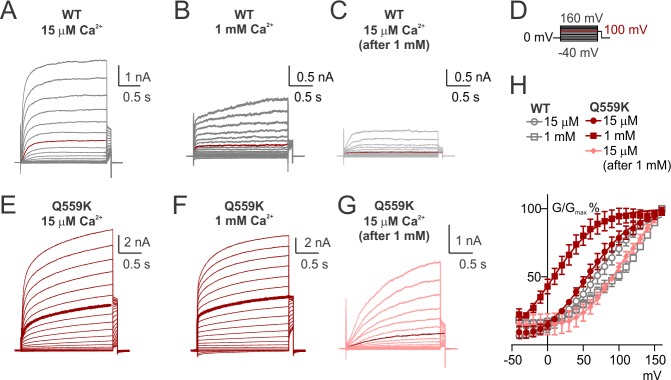
Voltage-dependence of TMEM16F WT and Q559K steady-state activation. (**A**, **B**, **C**, **E**, **G**, **F**) Representative traces recorded with a voltage family protocol from −40 mV to +160 mV with 10 mV increments followed by holding at +100 mV, as in (**D**). The traces recorded at +100 mV are highlighted for comparison. (**H**) Averaged G-V relationships of the currents recorded in indicated conditions. Tail current (at +100 mV) magnitudes were measured from traces as in *A* ~ *C, E ~ G*. For WT and Q559K in 15 µM Ca^2+^ and Q559K in 1 mM Ca^2+^. Tail current magnitudes of each recording were fitted to the Hill equation against voltage, and they were subsequently normalized to the respective maximal values. We chose to use Hill equation since it provides a simplified estimation for the kinetics of voltage activation (Yifrach, 2004). Averaged G/G_max_ values were calculated at the end for each group. For the other conditions, the tail current magnitudes were directly normalized to those following +160 mV for each recording, and the averaged G/G_max_ values for each group were calculated at the end.

**Figure 1—figure supplement 2.**
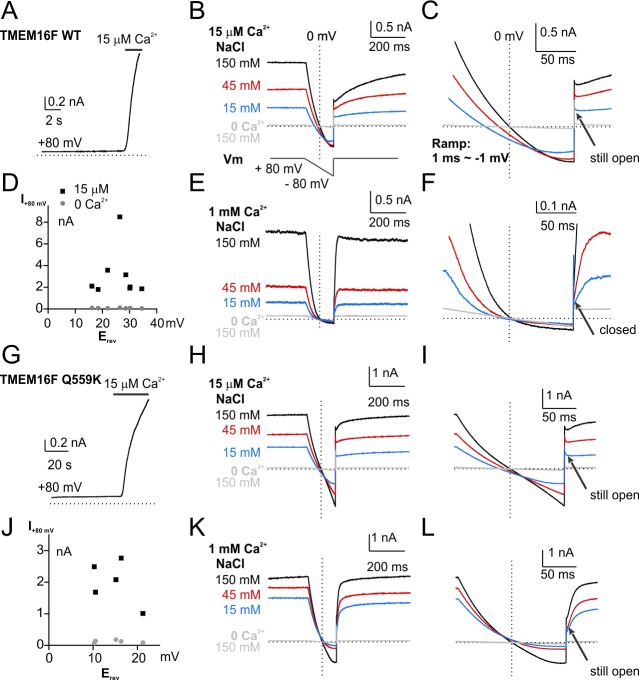
Recordings in indicated conditions with a hyperpolarizing ramp protocol. (**A,G**) Time courses of TMEM16F WT and Q559K activation by 15 µM Ca^2+^ in 150 mM NaCl. The currents were recorded when the patches were held at +80 mV. (**B, E, H, K**) Recordings of reversal potential with a hyperpolarizing ramp protocol following constant holding at +80 mV. For the demonstrated traces, the solutions were applied in the order of 150 mM, 45 mM and 15 mM NaCl, and back to 150 mM NaCl (not shown). Ca^2+^-free solution was applied at the end to confirm the seal. (**C, F, I, L**) Detail amplification showing tail currents in *B, E, H, K* recorded at +80 mV following the hyperpolarizing ramp. Note that in *F*, the tail current is not significantly bigger than endogenous current (gray trace), excluding the feasibility to calculate E_rev_ out of it. (**D, J**) Current magnitudes activated by 15 µM Ca^2+^ in 150 mM NaCl were plotted against the E_rev_ values measured with 15 mM NaCl intracellular solution, indicating that there is no correlation.

**Figure 1—figure supplement 3.**
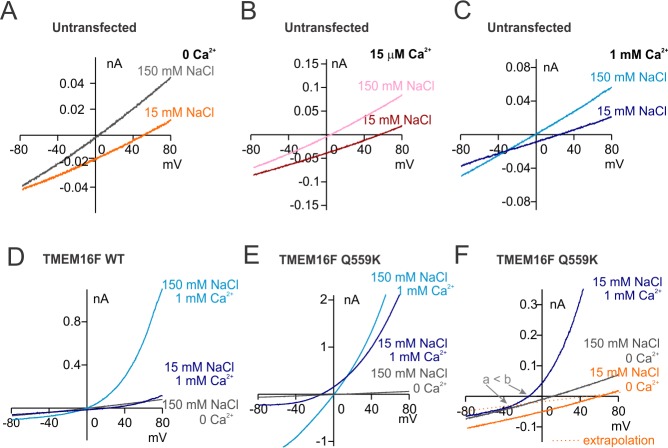
Additional control experiments for Figure 1. (**A ~ D**) Representative I-V relationships of currents recorded in indicated conditions. The recording protocol was the same as in Figure 1E,F. Wild-type TMEM16F current in 1 mM Ca^2+^ is not distinguishable from HEK293 cell endogenous currents. (**E**) Representative I-V relationships of TMEM16F Q559K current to show the E_rev_ in 1 mM Ca^2+^. Ca^2+^-free solution was applied at the end to confirm the seal. (**F**) One example of TMEM16F Q559K recorded in 1 mM Ca^2+^ to illustrate the challenge of background subtraction. Note that the trace of the current recorded in Ca^2+^-free 15 mM NaCl solution (orange solid trace) did not cross with that in 1 mM Ca^2+^ 15 mM NaCl (blue trace), attributable to the inhibition of endogenous current by 1 mM Ca^2+^, shown in *C*. Extrapolation (orange dotted trace) indicates that the current after background subtraction (**a**) should reverse at a more negative potential than that without subtraction (**b**), justifying our ‘safer’ step of not performing background subtraction (using b) for comparison involving recordings in 1 mM Ca^2+^.

**Figure 1—figure supplement 4.**
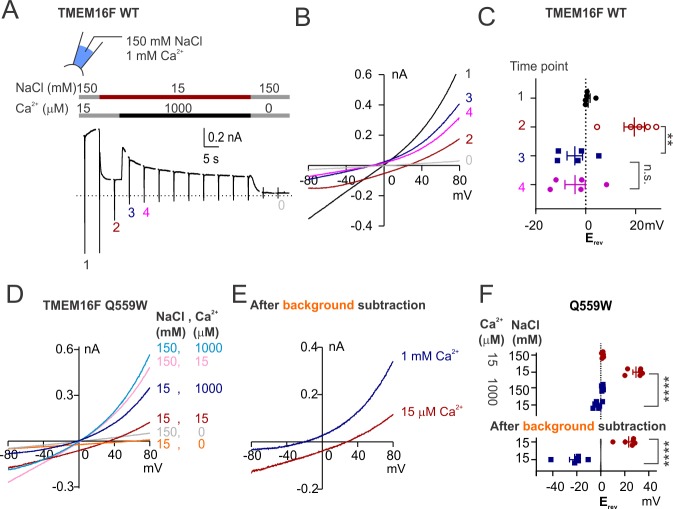
Permeability ratio P_Na+_/P_Cl-_ was altered in WT TMEM16F and Q559W in response to change of Ca^2+^. (**A**) Representative recordings of WT TMEM16F held at +80 mV with a hyperpolarizing ramp (−1 V/s) once every 5 s in indicated conditions. (**B**) Representative I-V relationships of currents recorded at labeled time points. (**C**) Scatter plot of the reversal potentials (E_rev_) at labeled time points without background subtraction. *p*-Values were determined with Tukey's multiple comparisons following one-way ANOVA. (**D**) Representative I-V relationships of Q559W recorded in indicated conditions. Traces were recorded with the same protocol as in Figure 1E,F. (**E**) I-V relationships of the currents in 15 µM Ca^2+^ and 1 mM Ca^2+^ (both in 15 mM NaCl) after background subtraction (subtraction of the current in Ca^2+^-free 15 mM NaCl solution, orange trace in *D*). (**F**) Scatter plot of reversal potentials (E_rev_) obtained from traces as in *D* (without background subtraction) and in *E* (after background subtraction). *p*-Values were respectively determined with Sidak's multiple comparisons following two-way ANOVA and two-tailed Student's *t*-test.

**Figure 1—figure supplement 5.**
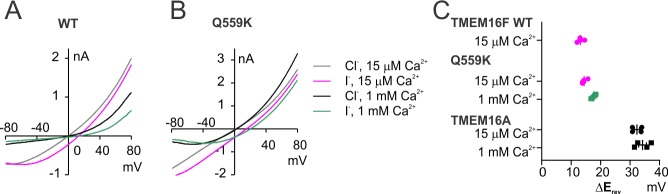
I^-^ permeability of TMEM16F WT and Q559K. (**A, B**) Representative I-V relationships of currents recorded in indicated conditions showing the shift of reversal potentials when the bath (intracellular) solution was switched from 150 mM NaCl to 150 mM NaI. (**C**) Scatter plot of the changes of reversal potentials (ΔE_rev_) when solution was switched from 150 mM NaCl to 150 mM NaI. WT currents in 1 mM Ca^2+^ were not included because they were not distinguishable from the currents endogenous to HEK293 cells. Because the interpretation methods for bi-ionic condition were not applicable here, we did **NOT** perform statistics and instead showed the values directly.

In contrast, the mutant Q559K current recorded at +80 mV showed minimal rundown in 1 mM Ca^2+^, in spite of desensitization to Ca^2+^ activation (Figure 1B) (Alvadia et al., 2019; Ye et al., 2018). Normalized to the respective maximal magnitudes of the currents before and after 1-min-treatment of 1 mM Ca^2+^, EC_50_ of Ca^2+^-activation was shifted from 5.6 ± 0.3 µM to 52 ± 11 µM (Figure 1C), while the normalized fully activated current magnitude was 0.89 ± 0.18 (current in 1 mM Ca^2+^ normalized to that before 1 mM Ca^2+^), significantly different from 0.36 ± 0.13 for wild type (Figure 1D). We also measured the steady-state conductance-voltage relationship from −40 mV to +160 mV. Desensitization caused a right-shift of the voltage-dependence of Q559K current in 15 µM Ca^2+^ (according to the comparison of 15 µM Ca^2+^-activated current before and after 1 mM Ca^2+^-treatment, Figure 1—figure supplement 1E,G), but in 1 mM Ca^2+^ the left-shift overrode the effect of desensitization, with V_1/2_ being 18 ± 10 mV (Figure 1—figure supplement 1F,H). The left shift of voltage-dependence in 1 mM Ca^2+^ probably explains the absence of rundown in Q559K current as observed for wild type, since at +80 mV Q559K in 1 mM Ca^2+^ is activated to a greater level than Q559K in 15 µM Ca^2+^ (with endogenous PIP_2_ in cell membrane) or wild type in 1 mM Ca^2+^ (Figure 1—figure supplement 1). Taken together, the Q559K mutation that minimized rundown allowed us to record TMEM16F current around physiological membrane potentials in a wide-range of Ca^2+^ concentration, here particularly, to measure E_rev_ in 1 mM Ca^2+^.

Interestingly, the E_rev_ of TMEM16F Q559K current activated by 1 mM Ca^2+^ exhibited a left-shift to −14 ± 1 mV in 15 mM NaCl, suggesting that it was more permeable to Cl^-^ than to Na^+^ (Figure 1F,G), in contrast to 12 ± 2 mV as the E_rev_ of it in 15 µM Ca^2+^ (recorded with the same protocol as for wild type above). Q559K mutant channels in 1 mM Ca^2+^ had a lower threshold for voltage activation than in 15 µM Ca^2+^ (Figure 1—figure supplement 1H), but the current quickly diminished with the hyperpolarizing voltage as in the case of wild-type TMEM16F (Figure 1—figure supplement 2K). The remaining current (confirmed with the ‘tail current’ recorded at +80 mV immediately following the ramp, Figure 1—figure supplement 2L) was nonetheless distinguishable from the background current endogenous to HEK293 cells, revealing that it reversed at a negative membrane potential. The current endogenous to HEK293 cells was partially inhibited in 1 mM Ca^2+^ (Figure 1—figure supplement 3A,B,C), thus rendering it difficult to measure for background correction in each recording in 1 mM Ca^2+^. However, with the confirmation that the endogenous current was selective for cation (Figure 1—figure supplement 3A,B,C) so that the ‘real’ TMEM16F-mediated current in 1 mM Ca^2+^ should reverse at an even lower membrane potential if the contribution of background current could be adequately removed (Figure 1—figure supplement 3F), we chose to take a ‘safer’ step by not performing background subtraction if the comparison involved currents activated by 1 mM Ca^2+^. Corrected for the liquid junction potential calculated with Clampex, these results indicated that the P_Na+_/P_Cl-_ was 0.47 ± 0.03 in 1 mM Ca^2+^, in contrast to 2.1 ± 0.4 as calculated from E_rev_ in 15 µM Ca^2+^.

We asked whether the phenotype of E_rev_ shifting is specific to the introduction of lysine to the position Q559 in TMEM16F. For the data shown above, E_rev_ measurements were made after the solution exchange was complete and the current alteration reached equilibrium, in which case the E_rev_ of wild-type TMEM16F in 1 mM Ca^2+^ was not measurable. Now we switched to 1 mM Ca^2+^ when the patch was incubated in 15 mM NaCl and immediately recorded E_rev_. With this protocol, we found a left-shift of E_rev_ accompanied with rapid current inactivation, until the current was too small to be distinguishable from endogenous current (Figure 1—figure supplement 4A,B,C). Thus, wild-type TMEM16F dynamically underwent the transition of an increased preference for Cl^-^ permeation with the elevation of intracellular Ca^2+^ concentration. It has been recently reported that mutation of Q559 to aromatic amino acids (such as Q559W) in TMEM16F also prevents current rundown (Nguyen et al., 2019). We measured the E_rev_ of Q559W current activated by 15 µM Ca^2+^ and 1 mM Ca^2+^ in 15 mM NaCl with the protocol as for Q559K. Although in each condition the E_rev_ of Q559W was more positive than that of Q559K, the E_rev_ shift persisted, regardless of background subtraction (with background currents recorded in Ca^2+^-free solutions with the same NaCl concentration) (Figure 1—figure supplement 4D,E,F). The above results showed that TMEM16F increased the relative permeability to Cl^-^ (versus Na^+^) when Ca^2+^ concentration was raised from 15 µM to 1 mM, in both wild type and mutants.

TMEM16F has been reported to be permeable to a variety of cations. With the respective published experimental settings, the permeabilities to many physiological cations (Na^+^, K^+^, Ca^2+^) were higher than that to Cl^-^, while the permeability to some other cations may be lower (Yang et al., 2012; Yu et al., 2015). Among anions, both wild-type TMEM16F and mutant channels are more permeable to I^-^ than Cl*^-^* (Figure 1—figure supplement 5) (Nguyen et al., 2019), similar to the preference of CaCC for larger anions (Schroeder et al., 2008; Caputo et al., 2008; Yang et al., 2008). In this study, we will mainly focus on the comparison between Na^+^ permeability and Cl^-^ permeability unless otherwise stated. Given the dynamic P_Na+_/P_Cl-_ ratio, we could not employ the calculation methods normally used for bi-ionic conditions to analyze data obtained with solutions involving a third ion species. Additionally, for each experiment in the following study, we performed paired-comparison within the same patches to avoid ambiguities arising from large variations across different recordings.

### Q559K channel permeability ratio P_Na+_/P_Cl-_ varies with intracellular Ca^2+^ concentration

**Figure 2.**
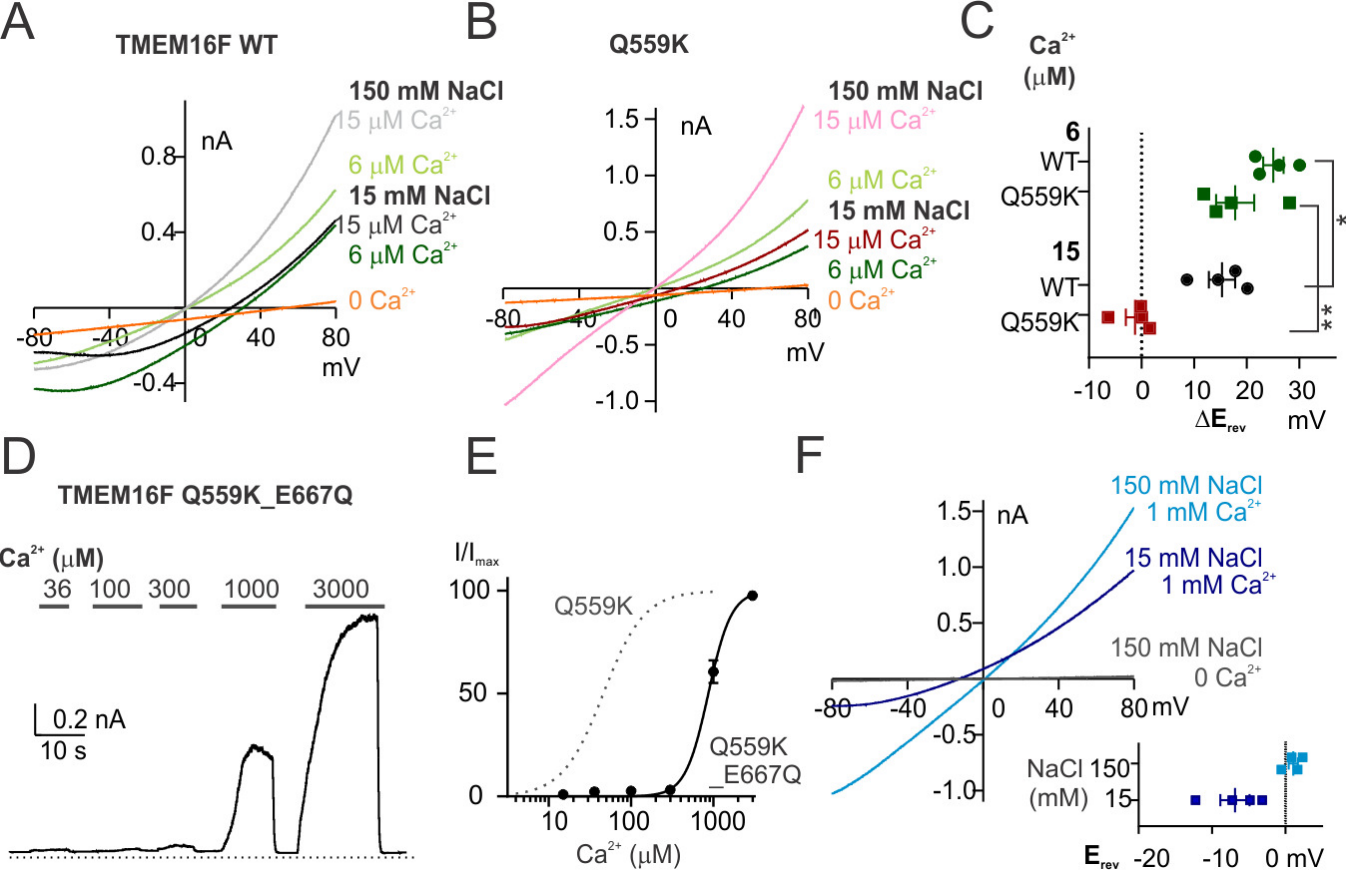
TMEM16F Q559K_E667Q channel is more permeable to Cl^-^ than to Na^+^ in 1 mM Ca^2+^despite being half activated. (**A, B**) Representative I-V relationships of WT and Q559K currents recorded in indicated conditions. The recording protocol was the same as in Figure 1E,F. Ca^2+^-free 15 mM NaCl solution was applied at the end for background subtraction. (**C**) Scatter plot of the changes of reversal potentials (ΔE_rev_) when solution was switched to 15 mM NaCl, obtained from traces as in *A* and *B. p*-Values were determined with Fisher's LSD test after two-way ANOVA. (**D**) Representative recordings of TMEM16F Q559K_E667Q in different Ca^2+^ concentrations. The recording protocol was the same as in Figure 1A. (**E**) Dose-response curve for Ca^2+^-activation of Q559K_E667Q. The gray dotted line represents the curve for Q559K after 1 mM Ca^2+^, replotted from Figure 1C. (**F**) Representative I-V relationships of Q559K_E667Q recorded in indicated conditions. The recording protocol was the same as in Figure 1E,F. The insert shows the scatter plot of reversal potentials (E_rev_) obtained from traces as in *F*.

We wondered whether the two types of ion selectivity of the Q559K mutant channel correspond to multiple open states. For example, the channel may be in an intermediate open state in 15 µM Ca^2+^ but a fully open state in 1 mM Ca^2+^. To test this possibility, we first tested the ion permeability ratio of current activated by 6 µM Ca^2+^, a condition where both WT and Q559K channels were activated to yield about half of the maximal current magnitudes (Figure 1A,B,C). To obtain accurate measurements, we subtracted the current recorded in Ca^2+^-free solution (with 15 mM NaCl) from those recorded in 6 and 15 µM Ca^2+^ for each excised patch. We found that for each recording from either WT or Q559K, the current evoked by 6 µM Ca^2+^ reversed at a more positive voltage than that by 15 µM Ca^2+^ (Figure 2A,B,C). To dissociate the effect of Ca^2+^ concentration from that attributable to different open states of the channel, we made use of the Ca^2+^-binding-site mutant, E667Q, that exhibits an elevation of EC_50_ of Ca^2+^-activation (Yang et al., 2012; Alvadia et al., 2019). The EC_50_ of the double-mutant, Q559K_E667Q, was 0.88 ± 0.06 mM, suggesting that the double mutant should not be in the fully open state in 1 mM Ca^2+^ (Figure 2D,E). If the increase of anion permeability only occurs when channel is fully open, the current through Q559K_E667Q channels should reverse at a positive potential in 1 mM Ca^2+^. Strikingly, the current in 1 mM Ca^2+^ reversed at −6.9 ± 2.0 mV, corresponding to a channel with higher permeability to Cl^-^ than to Na^+^ (Figure 2F). This indicates either that TMEM16F ion permeability preference is not correlated with its open state, or that the E667Q mutation circumvents the intermediate open state (if any) and causes the mutant channel to directly enter a fully-open conformation.

**Figure 3.**
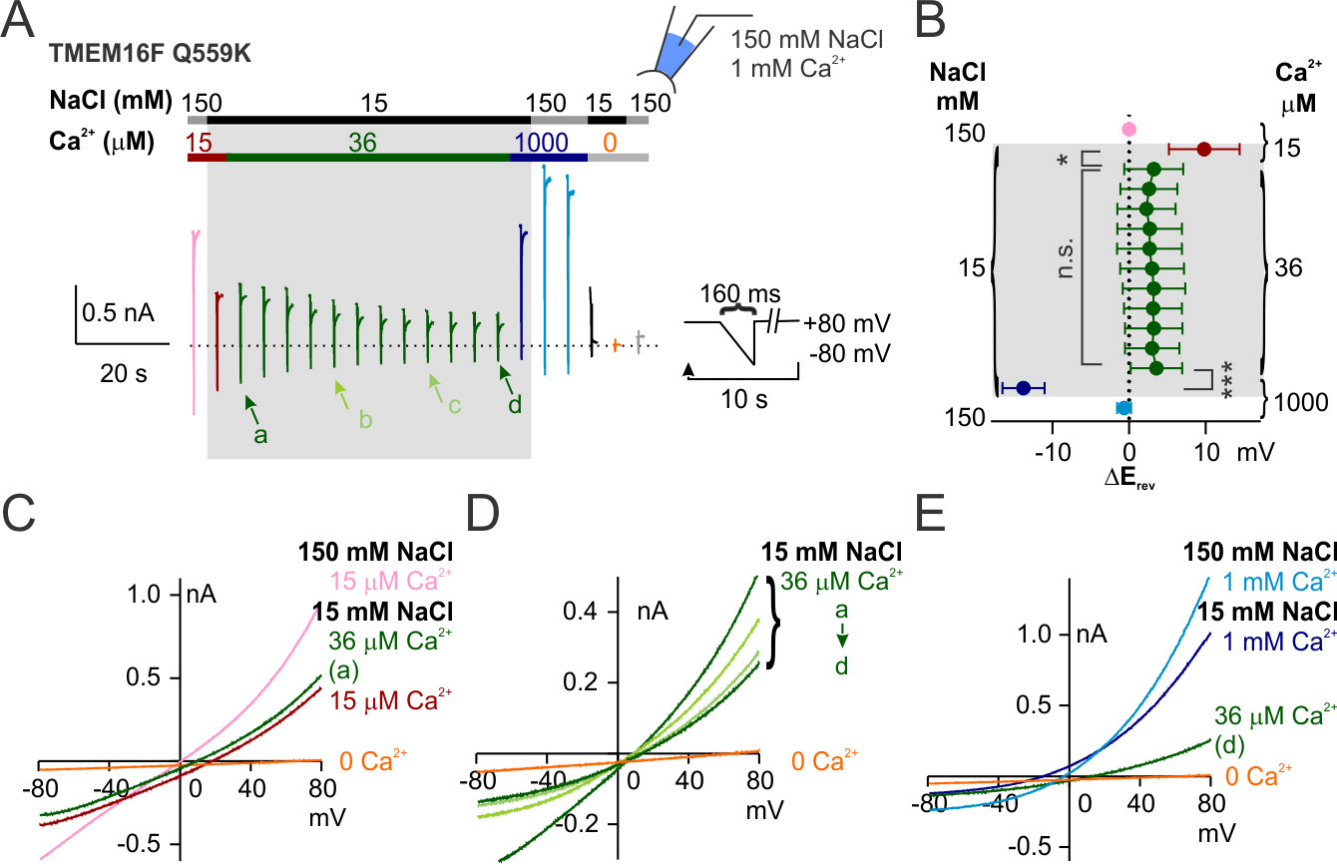
Q559K E_rev_ only shifts with the change of Ca^2+^ concentration. (**A**) Representative recording of TMEM16F Q559K held at +80 mV with a hyperpolarizing ramp (−1 V/s) once every 10 s in indicated conditions. (**B**) Summary of the averaged E_rev_ with background subtraction (subtraction of the current in Ca^2+^-free 15 mM NaCl solution, orange trace in *A*, except for the current in 1 mM Ca^2+^) in indicated conditions. For all the traces recorded in 15 mM NaCl, E_rev_ only shifts when Ca^2+^ concentration is changed. (**C, D, E**) Representative I-V relationships of Q559K at arrowed points in *A*, showing that E_rev_ shifts when Ca^2+^ is increased from 15 µM to 36 µM, persists in 36 µM Ca^2+^ despite the constant rundown, and shifts when Ca^2+^ is increased from 36 µM to 1 mM.

To dissociate the correlation between Ca^2+^ concentration and activation state, we utilized the desensitization phenotype of TMEM16F Ca^2+^-gating via exposure to high Ca^2+^. We previously reported that TMEM16F in 36 µM Ca^2+^ undergoes a slow desensitization with time constant of ~60 s possibly due to the slow degradation of PIP_2_. TMEM16F Q559K activation by 36 µM Ca^2+^ reached full or less-than-half open state, respectively, before or after desensitization (Figure 1B,C). We maintained the inside-out patches from Q559K-transfected cells in 36 µM Ca^2+^ (15 mM NaCl) for 90–100 s and measured E_rev_ once every 10 s (Figure 3). The E_rev_ was left-shifted when the Ca^2+^ concentration was raised from 15 µM to 36 µM. During the slow desensitization in 36 µM Ca^2+^, E_rev_ (corrected with subtraction of background current recorded in 0 Ca^2+^ afterwards) remained unchanged despite the constant decrease of current magnitude and the increase of outward rectification. We therefore concluded that the reversal potential of Q559K shifted only when Ca^2+^ concentration was changed, independently of current magnitude, open state, or rectification level. Also, although it was not clear whether the permeation pathway could be partially composed of the headgroups of lipids being scrambled, we inferred that at least PIP_2_, which was slowly depleted during incubation in 36 µM Ca^2+^, did not contribute to the shift of ion permeability ratio.

**Figure 4.**
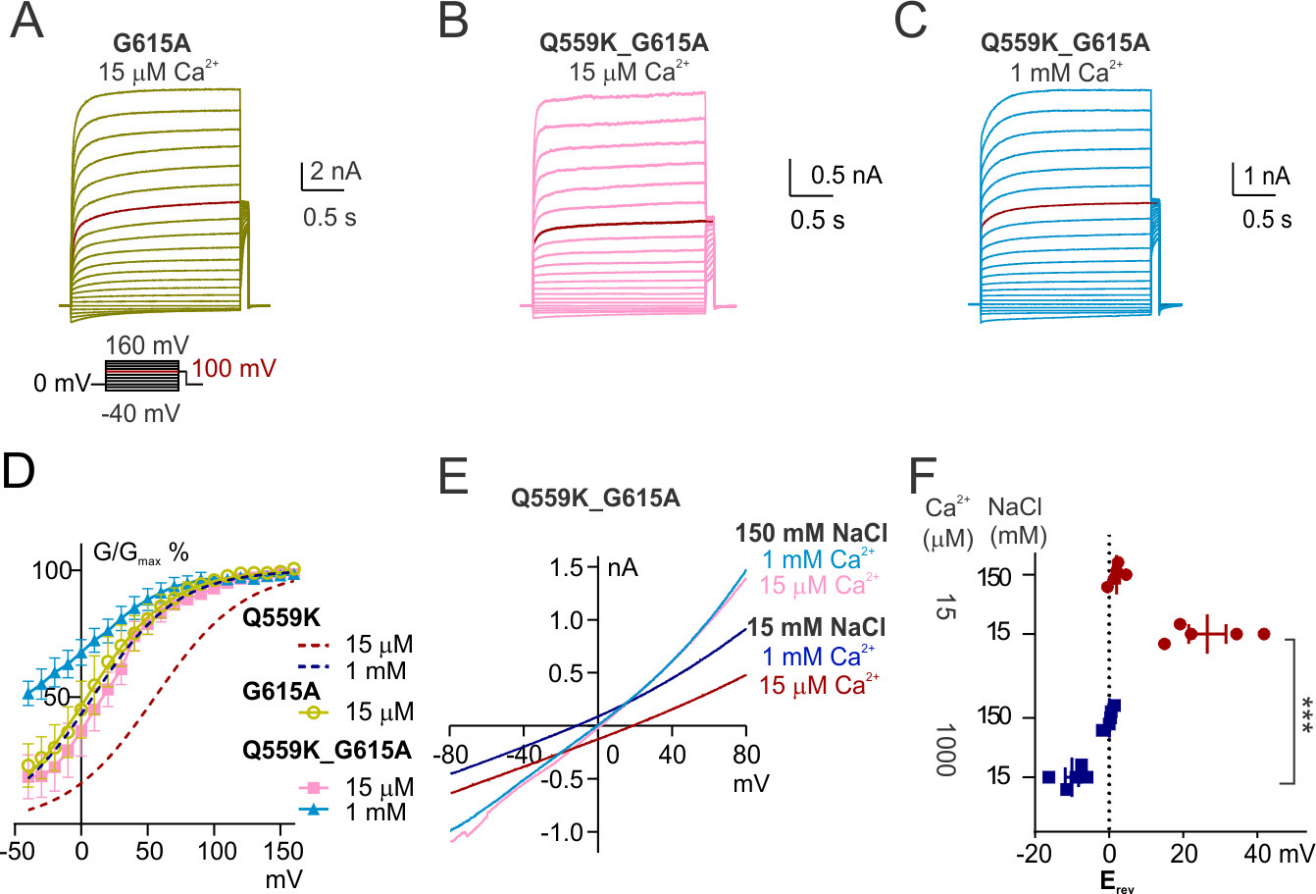
The change of ion permeability ratio is preserved despite TM6 conformational stabilization. (**A, B, C**) Representative traces of G615A and Q559K_G615A recorded with a voltage family protocol as in Figure 1—figure supplement 1. The currents recorded at +100 mV are highlighted for comparison. (**D**) Averaged G-V relationships of G615A and Q559K_G615A currents. The method for data analysis was the same as that for WT in 15 µM Ca^2+^. The two traces for Q559K were replotted from Figure 1—figure supplement 1H. (**D**) Representative I-V relationships of Q559K_G615A recorded in indicated conditions. (**F**) Scatter plot of reversal potentials (E_rev_) obtained from traces as in *E* without background correction. *p*-Values were determined with Sidak's multiple comparisons following two-way ANOVA.

We investigated whether the shift of relative ion permeability involves the conformational change of TM6, a critical step in channel activation. In TMEM16A, the glycine in TM6 (G640 or G644 depending on isoform) works as a hinge to allow the rearrangement of the helical segments during channel activation to generate the ionic permeation pathway (Paulino et al., 2017a; Peters et al., 2018). Alanine substitution of this glycine stabilizes TM6 at the open conformation and increases TMEM16A Ca^2+^ sensitivity (Paulino et al., 2017a; Peters et al., 2018). This glycine hinge in TMEM16A TM6 corresponds to G615 in TMEM16F according to sequence alignment (Figure 4—figure supplement 1A). Cryo-EM studies reveal that TM6 is bent at this glycine hinge in Ca^2+^-free but not Ca^2+^-bound TMEM16F (Feng et al., 2019). Previous studies have shown that its alanine substitution (G615A) increases Ca^2+^ sensitivity (Alvadia et al., 2019; Han et al., 2019), and we confirmed that G615A left-shifted the voltage dependence of channels recorded in 15 μM Ca^2+^ (Figure 4A,D). In contrast, mutation of the adjacent glycine, G614A, did not have similar or further effects (Figure 4—figure supplement 1E,F,G). We also confirmed the effect of G615A mutation by comparing its voltage-dependent activation with another two TM6 mutants, I612A and N620A, which were chosen because the alanine substitutions of their corresponding amino acids in TMEM16A (I637A or I641A, Q645A or Q649A, depending on isoform) represent the stabilization of the two steps of TM6 conformational rearrangement during TMEM16A activation, respectively (Peters et al., 2018). TMEM16F I612A, mutation of the isoleucine in TM6 upper segment, caused shifts in Ca^2+^- and voltage-gating by an extent comparable with those of G615A (Figure 4—figure supplement 1D,G) (Han et al., 2019), indicative of a relationship similar to that between TMEM16A G644A and I641A (amino acid labels using TMEM16A isoform as in Lam and Dutzler, 2018; equivalent to G640A and I637A for the isoform as in Peters et al., 2018 ), indicating that the two mutants might be stabilized in the same state. In the lower segment, TMEM16A Q649 (Q645 in Peters et al., 2018 ), whose alanine substitution facilitates channel opening (Peters et al., 2018; Lam and Dutzler, 2018), corresponds to a gap of the alignment in TMEM16F (Figure 4—figure supplement 1A); alanine substitution of the asparagine ‘5-amino-acid-away’ in TMEM16F TM6, N620, did not facilitate Ca^2+^ (Han et al., 2019) or voltage gating (Figure 4—figure supplement 1B,C). Taken together, mutation of G615 to alanine (G615A) might sufficiently stabilize TM6, which in wild-type TMEM16F undergoes rearrangement to generate the ionic permeation pathway.

**Figure 4—figure supplement 1.**
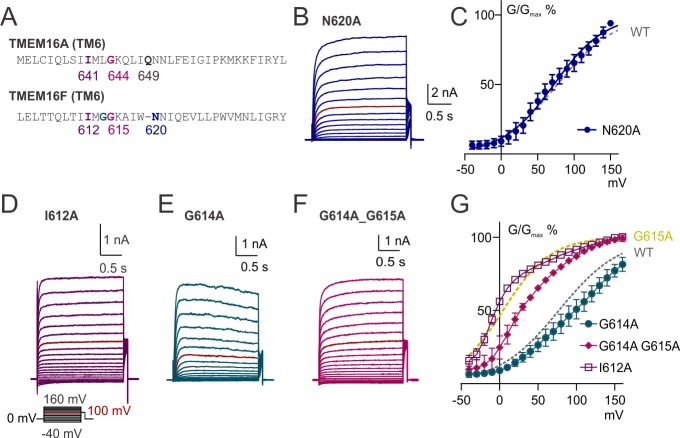
TM6 functions in TMEM16F gating. (**A**) Alignment of TM6 sequences of TMEM16A and TMEM16F. The numbering for TMEM16A represents the isoform as used by Lam and Dutzler (2018). (**B**, **D, E, F**). Representative traces of TMEM16F mutants recorded in 15 µM Ca^2+^ with a voltage family protocol as in Figure 1—figure supplement 1. Currents recorded at +100 mV are highlighted for comparison. (**C, G**) Averaged G-V relationships of indicated TMEM16F mutants. The method for data analysis was the same as that for WT in 15 µM Ca^2+^. The traces for WT and G615A were replotted from Figure 1—figure supplement 1H and Figure 4D.

**Figure 4—figure supplement 2.**
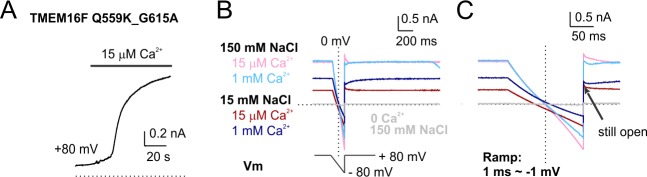
Recordings in indicated conditions with a hyperpolarizing ramp protocol. (**A**) Time courses of TMEM16F Q559K_G615A activation by 15 µM Ca^2+^ in 150 mM NaCl. The currents were held at +80 mV. (**B**) Recordings of reversal potentials with a hyperpolarizing ramp protocol following constant holding at +80 mV. The solutions were applied in the order of 150 mM, 15 mM NaCl with 15 µM Ca^2+^, 150 mM NaCl, 15 mM NaCl with 1 mM Ca^2+^. Ca^2+^-free solution was applied at the end to confirm the seal. (**C**) Detail amplification showing tail currents in *B* recorded at +80 mV following the hyperpolarizing ramp.

Notably, the steady-state voltage dependence of TMEM16F G615A channel in 15 μM Ca^2+^ was comparable to that of Q559K in 1 mM Ca^2+^ (Figure 4A,D). We then generated the double-mutant Q559K_G615A, which in 1 mM Ca^2+^ showed further left-shift of G-V relationship (Figure 4B,C,D). Recorded with the fast hyperpolarizing ramp, the current was almost linear, consistent with the notion that removal of the glycine hinge stabilizes the open conformation (Figure 4—figure supplement 2). The change of the permeability ratio P_Na+_/P_Cl-_ with rising Ca^2+^ in this double mutant was similar to that of Q559K (and wild-type) TMEM16F (Figure 4E,F). This double mutant revealed that in the presence of Q559K mutation, TMEM16F channel in 15 μM Ca^2+^ was modestly more permeable to Na^+^, but it became more permeable to Cl^-^ in 1 mM Ca^2+^ regardless of TM6 stabilization. Taken together, these results suggest that TMEM16F ion selectivity more likely depends on intracellular Ca^2+^ concentration directly rather than its open state(s), although it remains possible that TMEM16F might undergo miniscule conformational changes at different Ca^2+^ concentrations that contribute to the transition of relative permeability.

### The shift of permeability ratio correlates with electrostatic change in permeation pathway

Intrigued by the possibility that Ca^2+^ alters anion accessibility to the TMEM16A pore through electrostatic effect (Lam and Dutzler, 2018), we hypothesize that the shift of permeability ratio of TMEM16F Q559K in response to elevation of intracellular Ca^2+^ is due to a change of electrostatic field along its permeation pathway. In this scenario, the effect ought to be elicited not only by Ca^2+^ but also by other divalent or trivalent cations (Lam and Dutzler, 2018). We chose to test Zn^2+^ and Gd^3+^, divalent and trivalent cations that are smaller in ionic radius than Ca^2+^ but capable of activating TMEM16F. Both Zn^2+^- and Gd^3+^-activations of TMEM16F were coupled with rapid inactivation even at low concentrations (Figure 5—figure supplement 1A,D), precluding the possibility to accurately plot the dose-response curves. The exposure to divalent-free solution (with EGTA) for ~30 s to 1 min following channel inactivation by Zn^2+^ allowed the current to fully recover in magnitude (Figure 5—figure supplement 1B,C), suggesting that the rapid current rundown was not attributed to desensitization triggered by PIP_2_ degradation, although it is unclear whether Zn^2+^ can shield the negative charges in PIP_2_ headgroups to elicit a reversible rundown.

**Figure 5.**
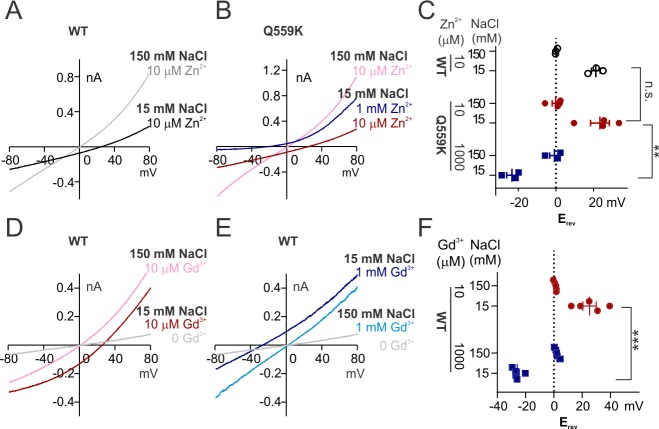
The change of ion permeability ratio is preserved when current is activated by Zn^2+^ or Gd^3+^. (**A, B**) Representative I-V relationships of WT and Q559K recorded in indicated Zn^2+^-containing solutions. The currents were recorded with the same protocol as in Figure 1E,F. (**C**) Scatter plot of reversal potentials (E_rev_) obtained from traces as in *A* and *B. p*-Values were determined with Sidak's multiple comparisons following two-way ANOVA. (**D, E**) Representative I-V relationships of WT TMEM16F recorded in indicated Gd^3+^-containing solutions. Note that the current in 1 mM Gd^3+^ 15 mM NaCl is still distinguishable from background current. (**F**) Scatter plot of reversal potentials (E_rev_) obtained from traces as in *E* and *F. p*-Values were determined with Sidak's multiple comparisons following two-way ANOVA.

We found that the wild-type TMEM16F channels activated by 10 μM Zn^2+^ were more permeable to Na^+^ than to Cl^-^, as evident from the right-shift in E_rev_ as intracellular NaCl dropped to 15 mM (Figure 5A). The E_rev_ in 1 mM Zn^2+^ could not be determined owing to the rapid current inactivation, so we again used the Q559K mutant to this end. Notably, TMEM16F Q559K channels exhibited an increased relative permeability to Cl^-^ (versus to Na^+^) with elevation of Zn^2+^ concentration from 10 μM to 1 mM (Figure 5B,C). In contrast to the drastic inactivation in 1 mM Zn^2+^, wild-type TMEM16F current activated by Gd^3+^, even with rundown, was still distinguishable from background current at both low and high concentrations. The remaining current underwent the E_rev_ shift when Gd^3+^ concentration was switched from 10 µM to 1 mM (Figure 5D,E,F). Note that wild-type TMEM16F current activated by Gd^3+^ showed weaker outward rectification (Figure 5E, compared with Figure 1—figure supplement 3D). However, the steady-state conductance-voltage relationship (Figure 5—figure supplement 1E,F) suggested that, instead of being activated independently of depolarization, the change of rectification was more likely due to a delayed channel closing during hyperpolarization.

**Figure 5—figure supplement 1.**
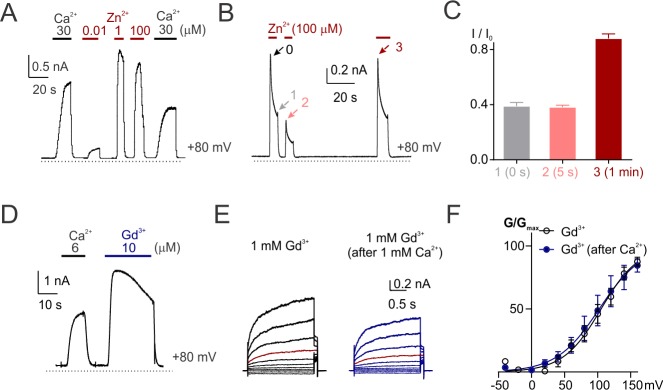
Supplementary recordings for Zn^2+^ and Gd^3+^activation of TMEM16F. (**A**) Representative recording of WT TMEM16F current in response to indicated concentrations of Zn^2+^ and Ca^2+^. The rundown of current in 1 µM Zn^2+^ was observed in all the three recordings. (**B**) Representative recording of WT TMEM16F current in response to multiple applications of Zn^2+^. (**C**) Averaged current magnitudes measured at arrowed time points normalized to the respective initial magnitudes (**I_0_**), indicating that Zn^2+^-inactivation is reversible. (**D**) Representative recording of WT TMEM16F current in response to indicated concentrations of Gd^3+^ and Ca^2+^. The rundown of current in 10 µM Gd^3+^ was observed in all the five recordings. (**E**) Representative traces of TMEM16F WT recorded in 1 mM Gd^3+^ with a voltage family protocol as in Figure 1—figure supplement 1. Currents recorded at +100 mV are highlighted for comparison. (**F**) Averaged G-V relationships of indicated TMEM16F WT activated by Gd^3+^. The method for data analysis was the same as that for WT in 15 µM Ca^2+^.

**Figure 6.**
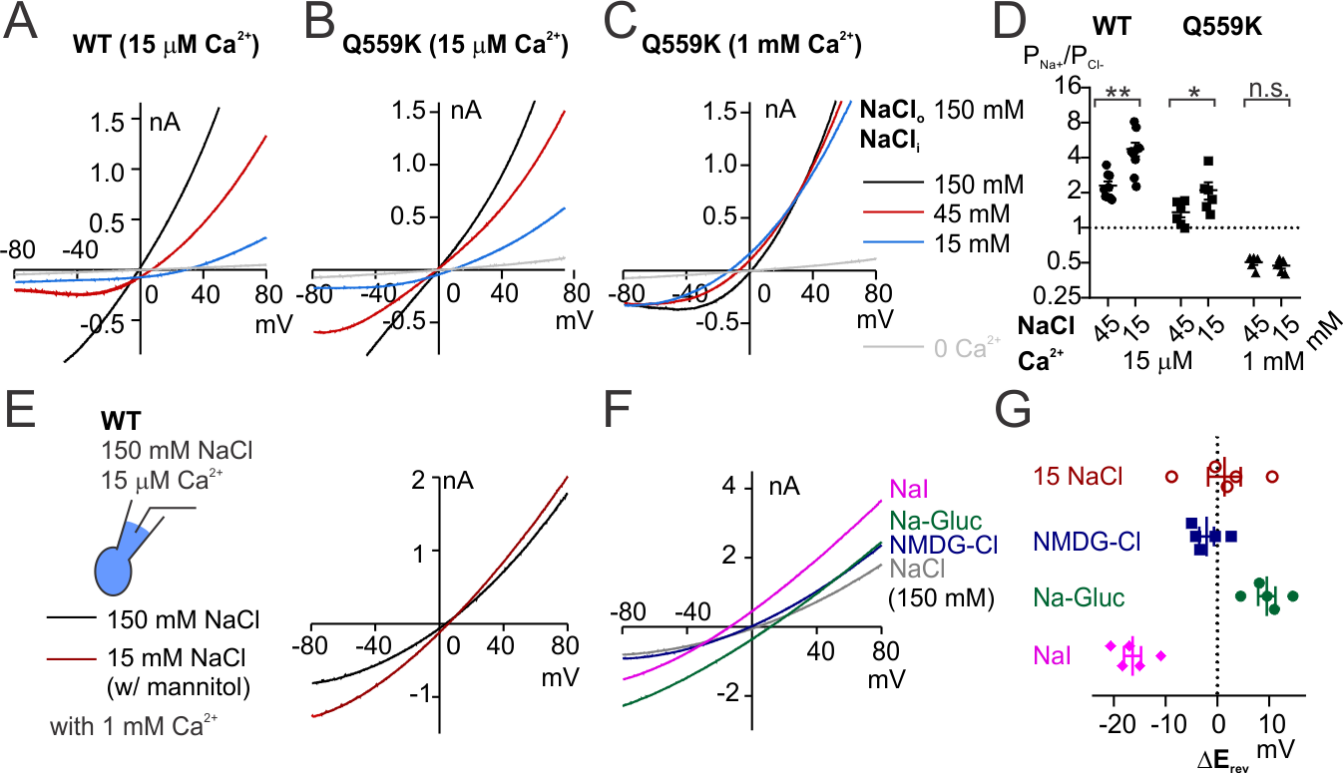
Ion permeability ratio is altered with change of intracellular NaCl concentration. (**A, B, C**) Representative I-V relationships of WT and Q559K recorded with inside-out configuration in indicated conditions. The currents were reanalyzed from the recordings as in Figure 1—figure supplement 2. Notice NOT to directly compare the shift of E_rev_ because the intracellular NaCl concentrations are varying. (**D**) Scatter plot showing the permeability ratio (P_Na+_/P_Cl-_) calculated from the shift of reversal potentials (ΔE_rev_) obtained from traces as in *A*, *B* and *C. p*-Value for WT was determined with Wilcoxon test. *P* values for Q559K were determined with Sidak's multiple comparisons following two-way ANOVA. (**E, F**) Representative I-V relationships of wild-type TMEM16F currents recorded with whole-cell configuration in indicated bath solutions. NMDG: N-methyl-D-glucamine; Gluc: gluconate. (**G**) Scatter plot of the changes of reversal potentials (ΔE_rev_) obtained from recordings as in *E* and *F*. Due to the potentially varying P_Na+_/P_Cl-_, we did NOT perform statistics or use them to calculate ion permeability ratios.

Although monovalent ions generate weak electrostatic fields compared with divalent and trivalent ions, given their abundance they should also be able to modulate the electrostatic field along TMEM16F permeation pathway and alter the relative ion permeability. TMEM16F is permeable to most generally-used cations including N-methyl-D-glucamine (NMDG) and tetraethylammonium (TEA) (Yang et al., 2012; Yu et al., 2015), so introducing any other ion species will cause difficulties in distinguishing whether the shift of E_rev_ is due to the change of P_Na+_/P_Cl-_ or to the permeation of the new ion species. Thus, we first calculated the respective permeability ratios from the measured E_rev_ with different intracellular NaCl concentrations (Figure 1—figure supplement 2). Compared with 15 mM NaCl, the stronger ionic strength of 45 mM intracellular NaCl enhances the screening effect for anions, and thus is predicted to increase the relative permeability to Cl^-^. Indeed, the calculated P_Na+_/P_Cl-_ values measured in 15 µM Ca^2+^ were 4.8 ± 0.6 and 2.3 ± 0.2 for wild-type TMEM16F in 15 mM and 45 mM NaCl, respectively; and the calculated P_Na+_/P_Cl-_ values measured in 15 µM Ca^2+^ were 2.1 ± 0.4 and 1.4 ± 0.1 for Q559K in 15 mM and 45 mM NaCl, respectively (Figure 6), consistent with previous findings that Q559K reduces P_Na+_/P_Cl-_ (Yang et al., 2012; Alvadia et al., 2019). The P_Na+_/P_Cl-_ for Q559K in 1 mM Ca^2+^ showed no significant difference between 15 mM NaCl and 45 mM NaCl (Figure 6), suggesting that millimolar Ca^2+^ outweighs Na^+^ in controlling the electrostatic effect. The shift of E_rev_ by changing the intracellular NaCl is also consistent with our proposed scenario that the shift of ion permeability ratio is not due to TM6 conformational change associated with different open states, but instead, due to the alteration of electrostatic field along the permeation pathway.

**Figure 6—figure supplement 1.**
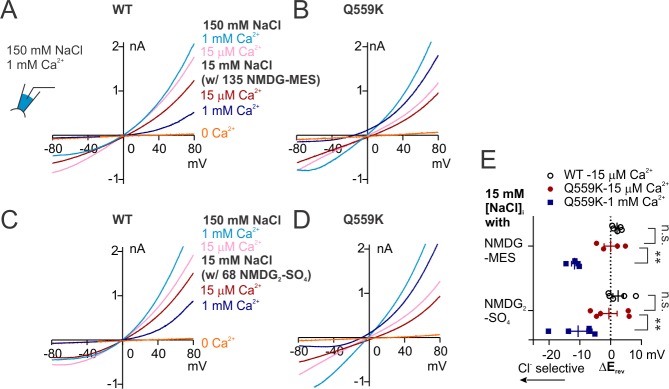
Recordings involving 15 mM NaCl bath balanced with NMDG-MES or NMDG_2_-SO_4_. (**A ,B, C, D**) Representative recordings of TMEM16F WT and Q559K in indicated bath conditions, where the osmolarity of 15 mM NaCl bath solution was balanced with NMDG-MES or NMDG_2_-SO_4_. The currents were recorded with the same protocol as in Figure 1E,F. Ca^2+^-free 15 mM NaCl (balanced with the respective salt composition) was applied at the end for background correction. Note that the E_rev_ values of WT currents in 1 mM Ca^2+^ (*A* and *C,* dark blue traces) were not distinguishable from those of the background currents (orange traces). (**E**) Scatter plot showing the changes of reversal potentials (ΔE_rev_) when solution was switched to 15 mM NaCl, obtained from traces as in *A ~ D*. Background subtraction was performed for currents activated by 15 µM Ca^2+^. *p*-Values were determined with Sidak's multiple comparisons following two-way ANOVA.

**Figure 6—figure supplement 2.**
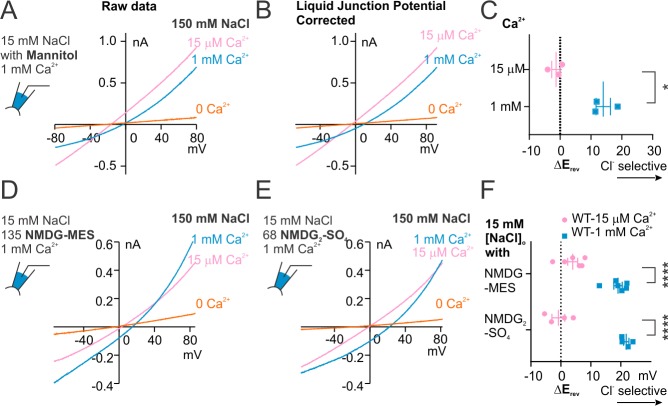
Recordings with 15 mM NaCl in the pipette solution. (**A**) Representative recordings of TMEM16F WT in different Ca^2+^ concentrations, where the pipette solution contained 15 mM NaCl balanced with mannitol. (**B**) Recording traces modified from A with liquid junction potential (12 mV) corrected. (**C**) Scatter plot of reversal potentials (E_rev_) obtained from traces as in (**B**). *p*- Value was determined with paired *t*-test. (**D, E**) Representative recordings of TMEM16F WT in different Ca^2+^ concentrations, where the pipette solution contained 15 mM NaCl balanced with mannitol NMDG-MES (**D**) or NMDG_2_-SO_4 _(**E**). The traces were plotted with the correction of liquid junction potentials (6 mM for *D* and 2 mM for *E*). (**F**) Scatter plot showing the reversal potential (E_rev_) after correction of liquid junction potentials, obtained from traces as in (**F**) and (**G**). *p*-Values were determined with Sidak's multiple comparisons following two-way ANOVA.

The minor shift of relative ion permeability by the change of intracellular NaCl concentration shown above might account for the discrepancy regarding TMEM16F ion selectivity recorded with various methods used by different labs. In the experiments above, the osmolarities of low NaCl solutions were all balanced with mannitol to avoid the interference by other ion species. Now, we tested for 15 mM NaCl with equal ionic strength as extracellular solution, which was balanced either with 135 mM NMDG-MES (MES: methanesulfonic acid) or 68 mM NMDG_2_-SO_4_. We observed that the wild-type TMEM16F activated by 15 µM Ca^2+^ in both conditions reversed at ~0 mV (2 ± 1 mV in 15 mM NaCl with NMDG-MES, 3 ± 2 mV in that with NMDG_2_-SO_4; _Figure 6—figure supplement 1A,C). Compared with E_rev_ recorded in 15 mM NaCl with mannitol, the reduction of E_rev_ might reflect a combination of NMDG permeation and the reduction of P_Na+_/P_Cl-_, assuming the permeation of MES^-^ or SO_4_^2-^ was negligible. In neither condition could we determine the E_rev_ for currents activated by 1 mM Ca^2+^. Using the mutant Q559K, we confirmed that in both conditions, the shift of E_rev_ with rising Ca^2+^ persisted (Figure 6—figure supplement 1B,D,E), which could be explained by an increase of P_Cl-_/P_Na+_ or a robustly increased permeation of NMDG, with the latter being less likely.

To confirm that the shift of ion permeability ratio does not depend on recording configuration, we altered the compositions of the pipette solution rather than those of the bath solution. We applied 15 mM NaCl balanced with mannitol, NMDG-MES or NMDG_2_-SO_4_ to the pipette solution (equivalent to extracellular solution), while the bath (equivalent to intracellular solution) contained 150 mM NaCl. With the correction of liquid junction potentials (12 mV, 6 mV and 2 mV respectively), the currents reversed at ~0 mV when activated by 15 µM Ca^2+^ in all the conditions (−1 ± 1 mV, 4 ± 2 mV and −1 ± 2 mV, respectively), while at 11 ± 2 mV, 19 ± 1 mV and 24 ± 1 mV, respectively, when activated by 1 mM Ca^2+^ (Figure 6—figure supplement 2). For the conditions involving NMDG, the E_rev_ shift either indicated an increased P_Cl-_/P_Na+_ or a robustly increased permeation of NMDG, with the latter being less likely. These results also complemented the previous tests involving changes of intracellular NaCl concentration (Figure 6A–D), showing that enhancement of intracellular ionic strength ‘unidirectionally’ promotes the relative permeability to Cl^-^. This can be attributed either to the preceding depolarization which drives cations into the pore, or to the possibility that the pore intrinsically adopts cations to modulate the electrostatic field. In addition, there was no significant difference in E_rev_ shifting between solutions balanced with NMDG-MES and that with NMDG_2_-SO_4_, suggesting that the divalent anions are relatively inert whether being applied intracellularly or extracellularly, attributable to the inaccessibility of divalent anions to the permeation pathway.

The above results suggest that TMEM16F ion selectivity is modulated by the change of electrostatic field along its permeation pathway, with divalent or trivalent cations having stronger impacts than monovalent ions. This might account for the lower selectivity for Na^+^ over Cl^-^ obtained from whole-cell recording, where intracellular solution was usually an isotonic salt solution. In whole-cell recording, TMEM16F current is reported to be elicited a few minutes after the whole-cell configuration is formed, and less selective for cations or in certain conditions even more selective for Cl^-^. Based on results reported above, we could infer that in these conditions the intracellular ionic strength (mainly maintained by ~150 mM NaCl or KCl) would reduce the channel relative permeability to cations. We performed whole-cell recording with 150 mM NaCl and 15 µM Ca^2+^ in the pipette, the E_rev_ was 1.4 ± 3.1 mV in 15 mM NaCl, indicative of P_Na+_/P_Cl-_ of 1.0 ± 0.1 (Figure 6D,F). Replacement of extracellular Na^+^ with NMDG did not significantly shift E_rev_, indicative of an increased permeability to Cl^-^ and/or a modest permeability to NMDG. Replacement of extracellular Cl^-^ with gluconate (Gluc) or I^-^ shifted E_rev_ to positive or negative values respectively, suggesting the presence of Cl^-^ conduction (Figure 6E,F) as observed in previous studies. However, we could not calculate the permeability ratios due to the potentially varying P_Na+_/P_Cl-_ in these conditions. Notably, it is also reported that *Tmem16f*-transfected cells undergo an ‘unconventional exocytosis’ which expands the membrane surface area dramatically during recording (Bricogne et al., 2019), leading to challenges in performing membrane capacitance compensation. These technical issues rendered it difficult to perform accurate measurements with whole-cell recording.

Taken together, the elevation of intracellular cation level underlies the increased permeability to Cl^-^, with divalent cations (Ca^2+^ and Zn^2+^) and trivalent cations (Gd^3+^) having stronger effects than monovalent ions (Na^+^ and NMDG). This suggests that TMEM16F employs a charge-screening mechanism to dynamically alter the preference for permeating ion species: Increase of positive charges, by introduction of basic amino acids or by entry of cations, enhances the channel preference for anions. This mechanism allows TMEM16F to open as a non-selective ion channel at low intracellular Ca^2+^ level and as a Cl^-^ channel when local Ca^2+^ concentration increases, indicative of physiological functions that have not been investigated thus far.

### Depolarization alters permeability ratio synergistically with intracellular Ca^2+^

**Figure 7.**
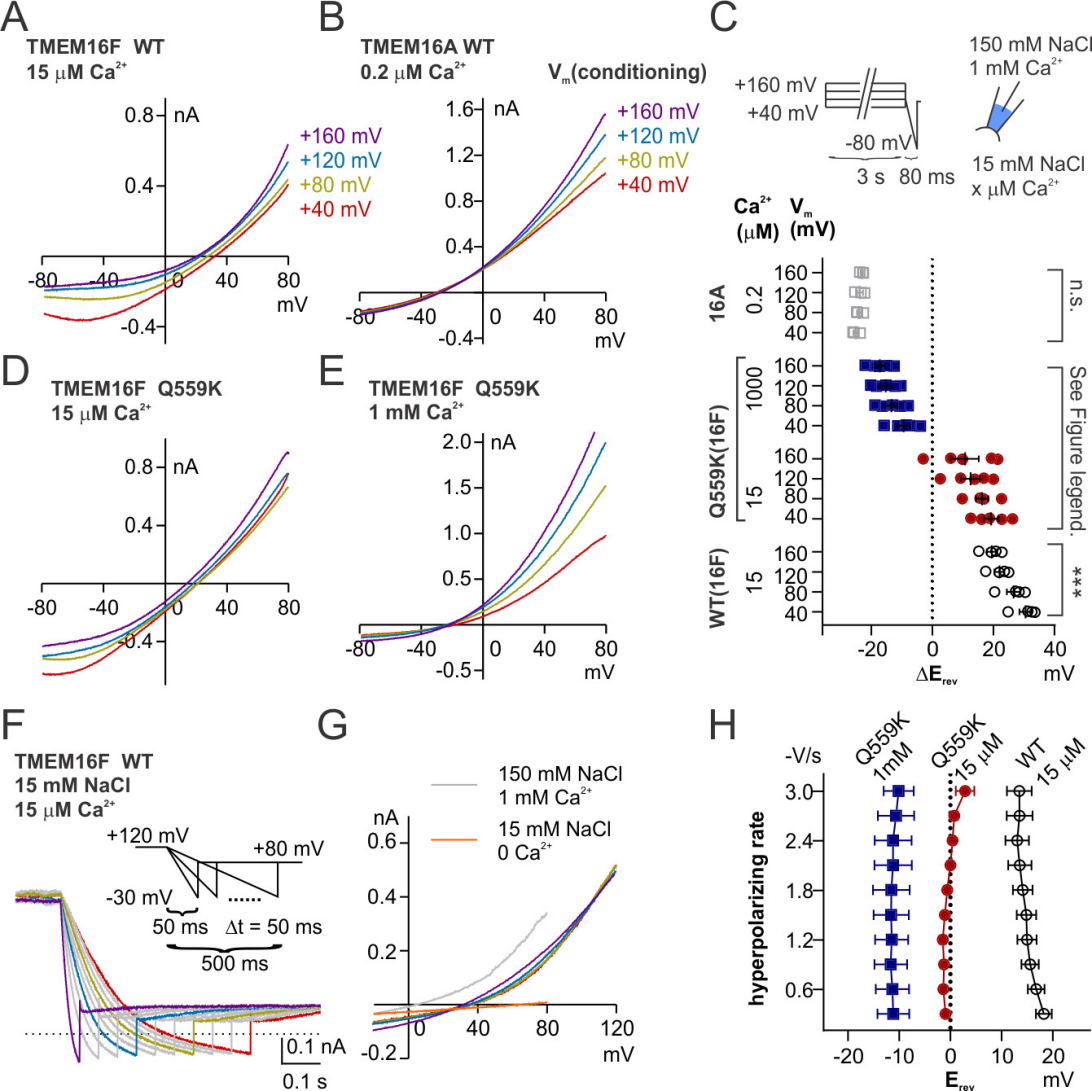
Depolarization alters permeability ratio synergistically with Ca^2+^ level. (**A, B, D, E**) Representative I-V relationships of currents recorded in indicated conditions. The excised patch was held at +40 to+160 mV with increments of 40 mV (‘conditioning potentials’) followed by a hyperpolarizing ramp from +80 mV to −80 mV (−2 V/s). (**C**) Scatter plot showing the changes of reversal potentials (ΔE_rev_) when solution was switched from 150 mM NaCl to 15 mM NaCl. For TMEM16A and WT TMEM16F, *p*-values were determined with one-way ANOVA. For TMEM16F Q559K, two-way ANOVA shows *p*<0.001 across voltages, *p*<0.0001 across Ca^2+^ concentrations. (**F**) Representative WT TMEM16F traces recorded in 15 µM Ca^2+^ 15 mM NaCl bath solution with the indicated protocol. The excised patch was held at 120 mV followed by a hyperpolarizing ramp from +120 mV to −30 mV (ramping speed from −0.3 V/s to −3 V/s), and the reversal potentials were corrected with background current recorded at the end. (**G**) The I-V relationships of the highlighted traces as in *F*, showing the currents reverse at the same point despite the change of rectification. (**H**). Summary of reversal potentials at different hyperpolarizing speeds obtained from traces as in *G*. Two-way ANOVA suggests that there is no significant difference among various hyperpolarizing speeds (*p*=0.28).

Previous studies have shown the synergy between Ca^2+^ and depolarization in the gating of TMEM16A. Depolarization facilitates Ca^2+^ entry into the membrane electric field and thus reducing the EC_50_ for channel activation by Ca^2+^ (Xiao et al., 2011; Peters et al., 2018). The reduction of EC_50_ of Ca^2+^ activation by depolarization is also observed in TMEM16F (Yang et al., 2012; Ye et al., 2018), although TMEM16F permeates Ca^2+^ (Yang et al., 2012), suggesting Ca^2+^ efflux is not fast enough to deplete Ca^2+^ from the electric field at the intracellular side. We asked whether depolarization also increases TMEM16F permeability to Cl^-^ by driving Ca^2+^ into the membrane electric field. To this end, we held the excised membrane patch at potentials ranging from +40 to+160 mV with increments of 40 mV (referred to as ‘conditioning potentials’) followed by a hyperpolarizing ramp from +80 mV to −80 mV (−2 V/s), to measure the E_rev_ (Figure 7C). With this experimental design, the shift of E_rev_ following different conditioning potentials could provide an indication of the changes of relative permeability, under the circumstance that during hyperpolarization TMEM16F can temporarily ‘memorize’ the ion permeability ratio at the preceding conditioning potential. We measured the E_rev_ of TMEM16F wild-type channels in 15 µM Ca^2+^, Q559K channels in 15 µM and in 1 mM Ca^2+^. In 15 µM Ca^2+^, the more depolarized the conditioning potential, the higher the relative Cl^-^ permeability (versus Na^+^ permeability) (Figure 7A,D). The traces recorded with the hyperpolarizing ramp following different conditioning potentials did not intersect with each other, confirming the E_rev_ alteration even though we did not perform background subtraction. The shift of E_rev_ was not a potential artifact caused by ion accumulation or depletion as previously investigated for other channels (Li et al., 2015; Yu et al., 2014), since if it was, a more depolarized conditioning potential would cause more severe Na^+^ accumulation or Cl^-^ depletion at the extracellular side (in the pipette solution), leading to a right-shift of E_rev_, which was opposite to our results. Traces of Q559K currents following different conditioning potentials in 1 mM Ca^2+^ overlapped at low membrane voltages (Figure 7E), a phenomenon observed for TMEM16A in 0.2 µM Ca^2+^ (Figure 7B). The gradual increase of relative Cl^-^ permeability caused by preceding depolarization can be attributed to intracellular cations, particularly Ca^2+^, to the membrane electric field to enhance the attraction of permeation pathway to anions, as can be inferred from the charge-screening mechanism.

**Figure 7—figure supplement 1.**
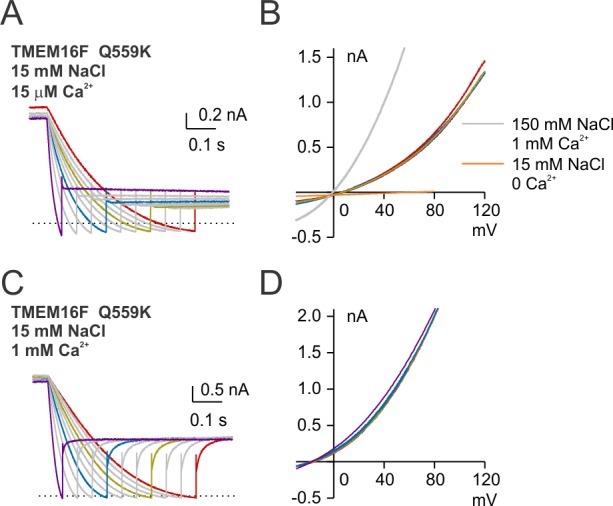
Representative traces of Q559K recorded with ramps of varoius hyperpolarizing speeds. (**A, C**) Representative TMEM16F Q559K traces recorded in 15 µM Ca^2+^ or 1 mM Ca^2+^, both with 15 mM NaCl bath solutions. The recording protocol was the same as in Figure 7F. (**B, D**) The I-V relationships of the highlighted traces as in *A* and *C*, showing the currents in each condition reverse at the same point.

The observation that TMEM16F in 15 µM Ca^2+^ is able to ‘memorize’ the ion permeability ratio at the preceding conditioning potential, suggests that the electrostatic field along the permeation pathway can be maintained even when there is no movement of net charge (i.e. 0 pA). We then recorded the E_rev_ with hyperpolarizing ramps of variable speeds (0.3 V/s to 3 V/s) following holding the patch at +120 mV, to test whether the channel would ‘forget’ the ion permeability ratio at the conditioning potential (+120 mV) by observing E_rev_s under hyperpolarization ramps of various rates. We did not see a significant shift of the E_rev_ for both WT and Q559K, although wild-type TMEM16F current became more outwardly rectifying at slower hyperpolarizing ramps (Figure 7F,G, Figure 7—figure supplement 1). Thus, the permeability ratio (P_Cl-_/P_Na+_) can be maintained during hyperpolarization, raising the possibility that the Ca^2+^ ion that modulates the pore electrostatic field dwells at the permeation pathway or a proximal site that allows it to still affect permeation pathway instead of leaving the pore with the ionic flow.

**Figure 8.**
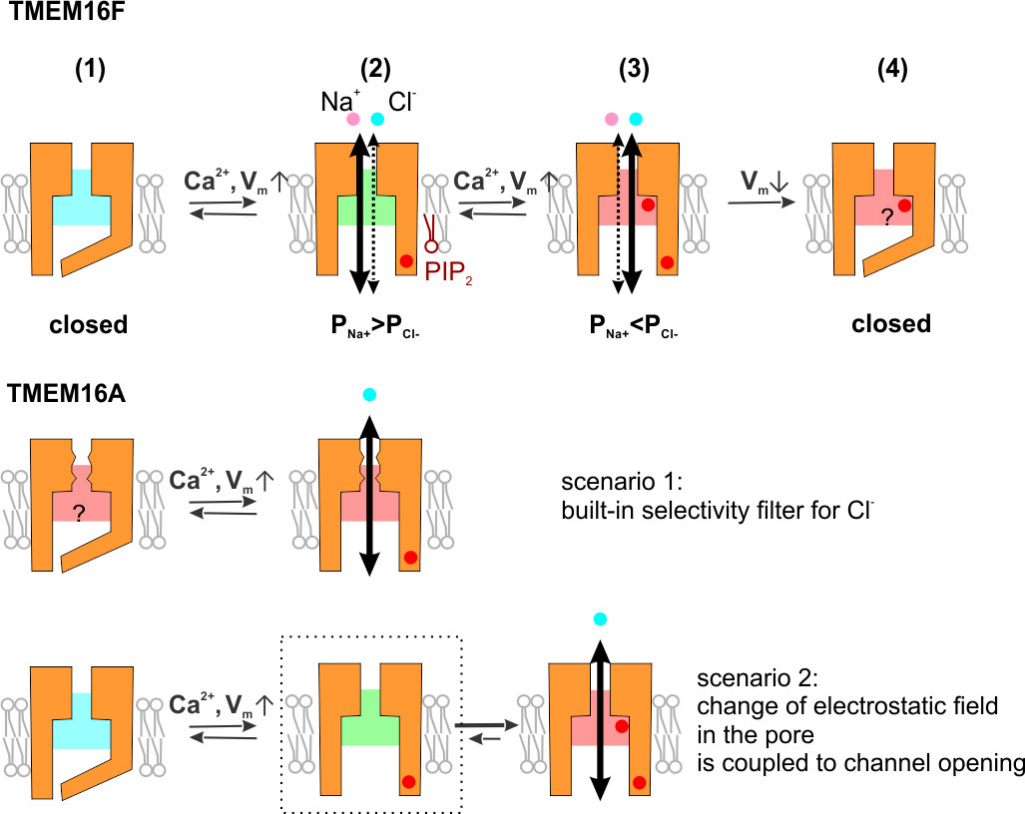
Diagram showing the proposed ion selectivity mechanism for TMEM16F and a comparison with TMEM16A. (**Upper**) TMEM16F gating and ion selectivity. The binding of intracellular Ca^2+^ to the binding-pocket triggers the rearrangement of transmembrane helices and opens the ion permeation pathway (from 1 to 2). The elevation of intracellular Ca^2+^ level shifts the electrostatic field along the permeation pathway from a Na^+^-favoring state (shaded with cold color) to that with increased attraction to Cl^-^ (shaded with warm color, from 2 to 3). Each red dot represents a Ca^2+^-entry event, while the number of Ca^2+^ ions for every event is not specified. Depolarization drives Ca^2+^ into both the binding pocket and the permeation pathway; PIP_2_ stabilizes the open conformation but does not affect the ion selectivity. Hyperpolarization triggers the closing of the channel, preceding the retreating of Ca^2+^ from the site that allows it to affect the permeation pathway, so that the channel maintains the electrostatic field and ‘memorizes’ the permeability ratio (from 3 to 4). (**Lower**) TMEM16A might harbor a selectivity filter restrictedly selective for anions (scenario 1), either with a size-dependent mechanism (illustrated with the zigzags) or a strong electrostatic field (illustrated with red shade). Alternatively, the change of electrostatic field might be tightly coupled to Ca^2+^-gating (scenario 2), so that the pore restrictedly selects for anions once it is opened.

We summarized the mechanism of TMEM16F dynamic ion selectivity based on the experiments shown above and published previously (Figure 8). TMEM16F ion permeation pathway is opened as a result of protein conformational change, which probably involves the rearrangement of TM6 induced by Ca^2+^-binding to the conserved Ca^2+^-binding pocket. The elevation of intracellular divalent or trivalent cation concentration brings positive charges into the permeation pathway, which gradually increases the relative permeability of anions. Depolarization promotes the pore attraction to anions by facilitating Ca^2+^ entry into the membrane electric field, wherein it may remain until the channel is closed.

**Figure 8—figure supplement 1.**
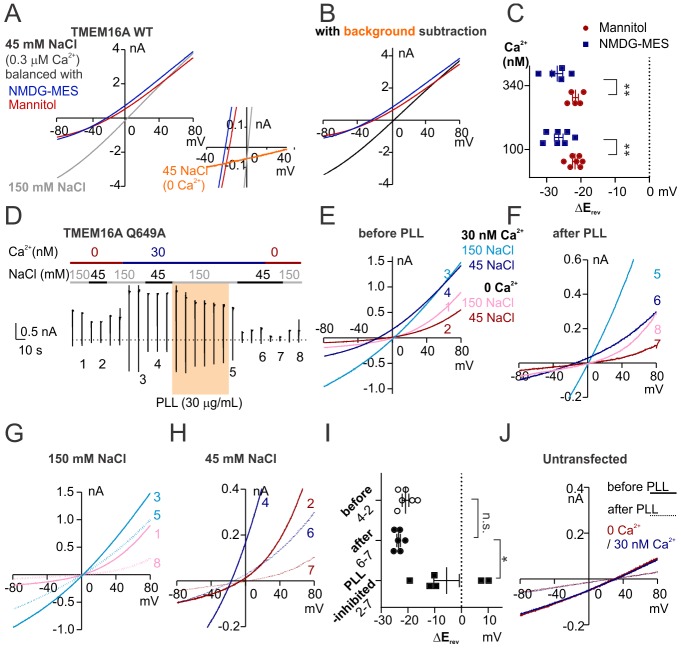
TMEM16A ion selectivity. (**A**) Representative I-V relationships of TMEM16A recorded in indicated conditions. Insert shows the detail amplification with the background current. (**B**) Representative I-V relationships of TMEM16A with background subtraction. (**C**) Scatter plot of E_rev_s obtained from traces as in *A,* with background subtraction. *p*-Values were determined with Sidak's multiple comparisons following two-way ANOVA. (**D**) Representative recording of TMEM16A Q649A held at +80 mV with a hyperpolarizing ramp (−1 V/s) once every 10 s under indicated conditions. The orange shade represents the treatment of poly-L-lysine (PLL) that promotes current rundown. (**E, F, G, H**) Representative I-V relationships of labeled traces as in *D*. (**I**) Scatter plot showing E_rev_s obtained from traces as in *D. p*-Values were determined with Tukey's multiple comparisons following one-way ANOVA. (**J**) Representative I-V relationships of an untransfected cell under indicated conditions, showing that PLL also triggers the rundown of the endogenous cation-selective current.

## Discussion

Studies regarding TMEM16F physiological functions have mostly focused on its phospholipid scrambling activities, while the understanding of its ion channel functions is confounded by the discrepancy of ion selectivity measurements. In cells with TMEM16F expression, increasing cytoplasmic Ca^2+^ concentration leads to activation of TMEM16F channel that mediates Ca^2+^ influx (Feng et al., 2019; Han et al., 2019), which further activates phospholipid scrambling. This is consistent with the results that TMEM16F recorded with inside-out excised patch showed higher permeability to cations than to Cl^-^ (Yang et al., 2012; Alvadia et al., 2019), notwithstanding the whole-cell recording indicating that delayed-activated TMEM16F is an anion channel (Grubb et al., 2013). In light of the Q559K mutant which displays minimal rundown and less outward rectification in 1 mM Ca^2+^, we measured TMEM16F reversal potentials at different Ca^2+^ concentrations, revealing a shift toward Cl^-^-selectivity at high intracellular Ca^2+^. This shift of ion permeability ratio correlates with the change of electrostatic field in the permeation pathway, which is determined by both intracellular ion concentration and membrane potential; divalent cations regardless of ion species are expected to exert stronger electrostatic effects than monovalent cations. This might reflect a general feature in the coupling of gating and conduction for channels in the TMEM16 family, likely relevant to their physiological functions.

### Gating and ion selectivity of TMEM16F

The ion permeation pathway of TMEM16F is generated probably through the rearrangement of TM6 in response to elevation of intracellular Ca^2+^ concentration, as reflected in its conformation in the presence or absence of Ca^2+^ (Feng et al., 2019), a mechanism similar to that of TMEM16A. Further elevation of intracellular Ca^2+^ results in an increase of relative permeability to Cl^-^ (versus Na^+^). With membrane potential and ionic strength unchanged, the permeability ratio P_Na+_/P_Cl-_ is only correlated with Ca^2+^ concentration rather than Ca^2+^-induced conformational change, suggesting that the ion selectivity machinery is not coupled to channel gating. At low Ca^2+^ when channels display modest selectivity between Na^+^ and Cl^-^, the ion permeability ratio is also altered by monovalent ions and membrane potential, which indicates that the ion selectivity filter is sensitive to electrostatic disturbance and thus might explain the discrepancy of experimental results from different labs. Depolarization, which drives Ca^2+^ into membrane electric field and facilitates Ca^2+^-gating, synergistically promotes the permeability alteration towards a preference for Cl^-^. The ion permeability ratio can be memorized during the recording with a hyperpolarizing ramp, suggesting that Ca^2+^ retreating from the site that affects the permeation pathway is preceded by the dissociation of Ca^2+^ from the binding pocket, so that the pore electrostatic field can be maintained until the channel is closed. PIP_2_ reduces the EC_50_ for Ca^2+^-activation but does not affect ion selectivity, thus facilitating TMEM16F opening when the pore is still more attractive to cations in low intracellular Ca^2+^. Controlling the selectivity between cations and anions by altering the pore potential, referred to as a charge-screening mechanism, is also documented in other ion channels, such as ligand-gated channels (Keramidas et al., 2004) and bacterial porins (Alcaraz et al., 2004), through charged residues lining the pore and/or through permeating ions within the pore.

It is intriguing to consider the structural basis for Ca^2+^ regulation of the TMEM16F relative ion permeability. Specifically, we wonder whether Ca^2+^ ion(s) in the Ca^2+^-binding pocket, or in the permeation pathway, may alter the electrostatic field of the pore. The former resembles what has been proposed for TMEM16A (Lam and Dutzler, 2018), whose pore is electrostatically modulated by Ca^2+^ bound to the pocket, but it seems unlikely for TMEM16F, because the Hill coefficient of Ca^2+^-activation of TMEM16F is ~2, suggesting that the two Ca^2+^-binding sites should both be occupied when the current displays higher permeability to cations. The latter scenario is that there is room for Ca^2+^-dwelling in the permeation pathway, consistent with the recently-published TMEM16F structure in which the pore harbors a large vestibule (Alvadia et al., 2019; Feng et al., 2019). Also, its distant homolog, TMEM16K, has multiple Ca^2+^ ions bound to its cytoplasmic domains (Bushell, 2018), raising the question whether there could be a reservoir in the TMEM16 cytoplasmic domain to store Ca^2+^. Given the long range electrostatic actions, it will be technically challenging to identify the structural basis for the electrostatic modulation in the pore.

### Comparison among TMEM16 members

TMEM16 represents a family of membrane proteins with diverse functions, including Ca^2+^-activated ion channel activities and Ca^2+^-activated lipid scrambling. Mammalian TMEM16A and TMEM16B are Ca^2+^-activated Cl^-^ channels (Schroeder et al., 2008; Caputo et al., 2008; Yang et al., 2008; Stephan et al., 2009), while other members with phospholipid scrambling functions display modest cation-selective or non-selective ion channel activities, such as TMEM16E (Whitlock et al., 2018), TMEM16F (Yang et al., 2012; Alvadia et al., 2019; Yu et al., 2015; Le et al., 2019a), *Drosophila* subdued (Le et al., 2019b), and two fungal TMEM16 homologs (Malvezzi et al., 2013; Brunner et al., 2014; Lee et al., 2016; Jiang et al., 2017). The lipid scrambling activity is also observed in an amoebozoa TMEM16 homolog (Pelz et al., 2018). All the TMEM16 proteins, based on reported structures and sequence alignment, are dimers with each monomer containing 10 transmembrane helices with conserved Ca^2+^-binding sites (Alvadia et al., 2019; Brunner et al., 2014; Paulino et al., 2017a; Bushell, 2018; Dang et al., 2017; Feng et al., 2019; Kalienkova et al., 2019; Falzone et al., 2019). Here, our studies reveal that TMEM16F displays a preference for Cl^-^ conduction when Ca^2+^ concentration is high, potentially indicative of a unifying mechanism regarding TMEM16 channel functions. In the following paragraphs, we will briefly discuss the similarities and differences among TMEM16 members, and focus mainly on the comparison between TMEM16A and TMEM16F.

In both TMEM16A and TMEM16F, the opening of the permeation pathway involves the conformational stabilization of TM6, while the removal of a glycine hinge reduces the Ca^2+^ level required for activation (Paulino et al., 2017a; Peters et al., 2018). Alanine substitution of the glycine reduces EC_50_s of Ca^2+^-activation in both TMEM16A and TMEM16F. However, TMEM16A and TMEM16F currents differ in rectification level (Nguyen et al., 2019). The current of TMEM16A bound to one Ca^2+^ ion (at low intracellular Ca^2+^ level) is outwardly rectifying, while at high intracellular Ca^2+^ level that allows two Ca^2+^ ions to occupy the binding-pocket, the TMEM16A channel conductance becomes ‘Ohmic’ (Peters et al., 2018). Such a transition can be explained by two mutually compatible mechanisms: the second Ca^2+^ ion triggers the further rearrangement of TM6 (Peters et al., 2018) and its positive electrostatic field reduces the energy barrier that intracellular anions need to overcome to access the permeation pathway (Lam and Dutzler, 2018). In contrast, TMEM16F current is outwardly rectifying in a wide range of Ca^2+^ concentration. It is important to keep in mind that TMEM16F rectification indices obtained from the traces recorded with ramp protocols (such as in Figure 1E,F) can be confounded with the voltage-dependent gating. However, according to the ‘instantaneous rectification index’ (Alvadia et al., 2019), which describes the magnitude ratio of outward current to inward current within the time range only allowing minimal change of gating, the TMEM16F pore is intrinsically outwardly rectifying, and wild type and Q559K channels are likely rectifying to a similar extent (Alvadia et al., 2019). Thus, in contrast to the corresponding pore-lining residue K588 in TMEM16A, which electrostatically interacts with the permeating Cl^-^ and is necessary for the ohmic conductance (Paulino et al., 2017b; Nguyen et al., 2019), the reduction of outward rectification of TMEM16F Q559K mutation might result from the alteration of voltage gating. Interestingly, the TMEM16F current activated by 1 mM Gd^3+^ is potentially approaching the ‘ohmic’ state (Figure 5E), suggesting that the strong electrostatic field of a trivalent cation bound to the protein could enhance the accessibility of intracellular Cl^-^ to the pore.

We propose two scenarios to explain why TMEM16F displays a shifting ion selectivity, while TMEM16A selects for Cl^-^ at all Ca^2+^ concentrations (Figure 8). In one scenario, TMEM16A has a ‘built-in’ selectivity filter that favors anions, so that only anions can eventually permeate even though both anions and cations can access the pore. Such a ‘built-in’ selectivity filter can either be a size-based screening structure, or a constantly strong positively-charged field to attract anions and repel cations. In the second scenario, TMEM16A couples the change of the electrostatic field to the opening of the pore, in a way that the former event precedes or synchronizes with the latter. We tried to distinguish between the two scenarios by measuring the ion selectivity of TMEM16A activated by low intracellular Ca^2+^. Notably, TMEM16A current activated by 100 nM Ca^2+^ or 300 nM Ca^2+^ is Cl^-^-selective, with the selectivity measured in low NaCl balanced with NMDG-MES being more selective for Cl^-^ (compared with Na^+^) than that in low NaCl balanced with mannitol (Figure 8—figure supplement 1A,B,C), a phenomenon consistent with a previous report (Lim et al., 2016). To probe TMEM16A ion selectivity in 0 Ca^2+^, we also generated the Q649A mutant of TMEM16A, which is activated by depolarization in Ca^2+^-free solution (Peters et al., 2018; Lam and Dutzler, 2018), and we used poly-L-lysine (PLL) to strip off membrane-tethered PIP_2_ to accelerate current rundown (De Jesús-Pérez et al., 2018). We noticed that TMEM16A Q649A activated by 30 nM Ca^2+^ was selective for Cl^-^ before or after PLL application, but the ‘rundown’ component (current in 0 Ca^2+^) displayed a dispersed distribution of E_rev_ (Figure 8—figure supplement 1D–I). Because PLL also triggered the rundown of the background current endogenous to HEK293 cells, which is cation selective (Figure 8—figure supplement 1J), we could not draw a definitive conclusion at this point.

### Functional implication

The shift of relative ion permeability with elevation of Ca^2+^ concentration may dynamically regulate membrane potential of the cell. Since TMEM16F can function as a Ca^2+^-activated Ca^2+^-permeable channel, the dynamic increase of Cl^-^ permeability (versus cation) might provide a brake to the positive feedback to reduce the influx of cations including Ca^2+^. In a typical neuronal cell, such a transition towards Cl^-^ selectivity might reduce the entry of Ca^2+^ into the cell and drive membrane potential close to Cl^-^ equilibrium potential, both of which will reduce the neuronal excitability and prevent Ca^2+^-overloading. The ‘memory’ of the permeability ratio during repolarization allows TMEM16F to modulate the waveform of action potentials in accordance to local cytoplasmic Ca^2+^ level. In non-excitable cells, this function might enable TMEM16F to sense cellular Ca^2+^ level and modulate membrane potential, which affects cellular proliferation and differentiation (Sundelacruz et al., 2009). It has also been reported that TMEM16F is localized in primary cilia (Forschbach et al., 2015), a special cellular compartment with elevated Ca^2+^ level and greater depolarization (Delling et al., 2013), providing a condition for TMEM16F to function dynamically. With the broad expression pattern of TMEM16F, we envision a new perspective to examine the functions of TMEM16F.

## Materials and methods

### Cell culture and molecular biology

Mouse *Tmem16f and Tmem16a* cDNAs (sequences as in NCBI RefSeq NM_175344.4, *Mus musculus Ano6*-splice variant 2, and NM_178642.5, *Mus musculus Ano1*-splice variant 1, respectively) were respectively fused with mCherry and subcloned into pcDNA3, as previously reported (Ye et al., 2018). Site-directed mutagenesis was performed using standard molecular techniques with pHusion polymerase (New England Biolabs, Ipswich, MA) and sequences were all verified (Quintara Biosciences, South San Francisco, CA). HEK (Human embryonic kidney)−293 cells (ATCC, RRID: CVCL_0045) were aliquoted and preserved in liquid nitrogen. One vial of cells was thawed and plated in 10-mL cell culture flask (ThermoFisher Scientific, Waltham, MA) once every 3 months. Cell were cultured in Dulbecco's Modified Eagle Medium (DMEM, with 4.5 g/L glucose, L-glutamine and sodium pyruvate, Mediatech, Manassas, VA) containing 10% FBS (Axenia BioLogix, Dixon, CA) and 1% penicillin-streptomycin, at 37°C and with 5% CO_2_, and they were passaged into a new flask with a 1:10 ratio once every 3 ~ 4 days. They were disposed of if they were not confluent in the flask in 4 days, their morphologies deviated from pictures in instructions (ThermoFisher website), or if the batch had been used for 3 months, whichever coming first, but were not in particular tested for mycoplasma contamination. Cells for electrophysiology recording were plated on 12 mm round coverslip (Warner Instruments, Hamden, CT) during the passaging procedure. Transient transfection was performed with Lipofectamine 2000 (ThermoFisher Scientific, Waltham, MA) 2 days before recording. The cDNA constructs for wild-type TMEM16F-mCherry, the mutants Q559K-, G615A-, G614A-, I612A-, G614_G615A-, N620A-, and E667Q- mCherry were stably transfected in HEK293 cells as previously reported (Han et al., 2019). Briefly, the cDNAs were subcloned into pENTR1A (Addgene plasmid #17398) and transferred to pLenti CMV Hygro DEST (Addgene plasmid #17454) using Gateway cloning (Campeau et al., 2009). pENTR1A no ccDB (w48-1) and pLenti CMV Hygro DEST (w117-1) were gifts from Dr. Eric Campeau and Dr. Paul Kaufman (University of Massachusetts Medical School, Worcester, MA, USA). TMEM16F-mCherry pLenti was co-transfected into HEK293FT cells with packaging plasmids pMD.2G and psPAX2, which were gifts from Didier Trono (Addgene plasmids # 12259 and #12260). Lentivirus was harvested from the transfected cells 36–48 hr post-transfection and incubated with HEK293 cells to establish stable cell lines under hygromycin selection.

### Solutions

For all electrophysiology recordings, bath solution contained 145 mM NaCl, 10 mM HEPES, 2 mM CaCl_2_, 1 mM MgCl_2_, 10 mM glucose, pH 7.2 with NaOH. For inside-out recordings, pipette solution contained 150 mM NaCl, 10 mM HEPES, 1 mM CaCl_2_, unless otherwise stated. The membrane patch was excised to form inside-out configuration in Ca^2+^-free solution: 150 mM NaCl, 10 mM HEPES, 2 mM EGTA. For solutions with Ca^2+^ < 100 µM, Ca^2+^ was added to solutions containing 2 mM EGTA or 2 mM HEDTA, and the final Ca^2+^ concentration was confirmed with Fluo-3 or Oregon Green BAPTA 5N (ThermoFisher Scientific). For whole-cell recording, the pipette solution contained 150 mM NaCl, 10 mM HEPES, 5 mM HEDTA and 4.1 mM CaCl_2_, and the final free Ca^2+^ concentration (15 µM) was confirmed with Oregon Green BAPTA 5N. The osmolality of each solution was adjusted to 290 ~ 310 mOsm/kg.

Solutions with NaCl lower than 150 mM were made by mixing the above solutions (if Ca^2+^ < 100 µM) with isotonic mannitol solution (300 mM mannitol, 10 HEPES), NMDG-MES solution (150 mM NMDG, 150 mM MES, 10 mM HEPES), or NMDG_2_-SO_4_ solution (150 mM NMDG, 75 mM H_2_SO_4_, 10 mM HEPES). Ca^2+^ concentration was subsequently confirmed with Oregon Green BAPTA 5N. The 1 mM Ca^2+^ solutions were made by directly mixing the solution containing 150 mM NaCl, 10 HEPES with the respective isotonic solutions, and Ca^2+^ was added at the end. Solutions in Figure 6F contained 150 mM NaI, Na-gluconate or NMDG-Cl with 10 mM HEPES, 1 mM Ca^2+^. Zn^2+^ and Gd^3+^ were directly added to solution of 150 mM NaCl, 10 HEPES from 100 mM stocks. The pH values of all the solutions were confirmed to be ~7.3. All the chemicals were purchased from Sigma-Aldrich (St Louis, MO).

### Electrophysiology

Cells were lifted with trypsin-EDTA (Life Technologies, Carlsbad, CA) and plated onto 12 mM coverslip (Warner Instruments, Hamden, CT) 3 ~ 4 days before recording. For recording, coverslips with cells were transferred to a chamber on a Nikon-TE2000 Inverted Scope (Nikon Instruments, Melville, NY) and transfection was confirmed with fluorescent microscopy. Patch borosilicate pipets (Sutter Instrument, Novato, CA) were pulled from a Sutter P-97 puller with resistances of 2–3 MΩ for inside-out patch recordings. Solutions were puffed to the excised patch using VC3-8xP pressurized perfusion system (ALA Science, Farmingdale, NY). Data were acquired using a Multiclamp 700B amplifier controlled by Clampex 10.2 via Digidata 1440A (Axon Instruments, Sunnyvale, CA). All experiments were performed at room temperature (22–24°C).

For measurements of reversal potentials, a 3 M KCl salt bridge was used. For experiments with 150 mM NaCl in the pipette solution, based on prediction by Clampex (Molecular Devices, Sunnyvale, CA), the liquid junction potentials for 15 mM NaCl, 45 mM NaCl (balanced with mannitol) were −1.7 mV and −1.0 mV respectively and were only corrected for calculation of permeability ratios (P_Na+_/P_Cl-_). For recordings with 150 mM NaCl pipette solution, the liquid junction potentials for all the other conditions were within ±2 mV, and were not corrected in the figures. For recordings where the pipette solution contained 15 mM NaCl (balanced with mannitol, NMDG-MES or NMDG_2_-SO_4_), the liquid junction potentials were corrected by adding 12 mV, 6 mV and 2 mV, respectively, to the measured values, as estimated by Clampex. The data in Figure 6—figure supplement 2B~F were displayed with the correction of liquid junction potentials.

### Data analysis

All data were analyzed using pClamp10 (RRID: SCR_011323, Molecular Devices, Sunnyvale, CA), OriginLab (OriginLab Corporation, Northampton, MA), and Graphpad Prism (RRID:SCR_002798, GraphPad Software, La Jolla, CA). For the measurement of Ca^2+^-sensitivity, every trace was fitted to the Hill equation to generate its respective EC_50_ and H (Hill coefficient). The curves in the figures display the averaged current magnitudes normalized to their respective maximal values (I/I_max_ %). P_Na+_/P_Cl-_ values were calculated with the simplified equation derived from Goldman-Hodgkin-Katz equation:

$$\Delta E_{rev}\;=\;59\;\times \;log\;[(P_{Na+}\times [Na^{+}{]}_{o}\;+\;P_{Cl-}\;\times [Cl^{-}{]}_{i})/(P_{Na+}\;\times \;[Na^{+}{]}_{i}\;+\;P_{Cl-}\;\times \;[Cl^{-}{]}_{o})],$$

where [Na^+^]_o_, [Cl^-^]_o_, [Na^+^]_i_, and [Cl^-^]_i_ are extracellular and intracellular Na^+^ and Cl^−^ concentrations, respectively.

Significant differences were determined with Student’s *t*-test and ANOVA unless otherwise stated. In all cases, data represent mean ± SEM, *p<0.05, **p<0.01, ***p<0.001, ****p<0.0001, n.s. *p*>0.05.

## Data Availability

All data generated or analysed during this study are included in the manuscript and supporting files.

## Additional files

Transparent reporting form

## References

[bib1] Alcaraz A, Nestorovich EM, Aguilella-Arzo M, Aguilella VM, Bezrukov SM (2004). Salting out the ionic selectivity of a wide channel: the asymmetry of OmpF. Biophysical Journal.

[bib2] Alvadia C, Lim NK, Clerico Mosina V, Oostergetel GT, Dutzler R, Paulino C (2019). Cryo-EM structures and functional characterization of the murine lipid scramblase TMEM16F. eLife.

[bib3] Bevers EM, Williamson PL (2016). Getting to the outer leaflet: physiology of phosphatidylserine exposure at the plasma membrane. Physiological Reviews.

[bib4] Bricogne C, Fine M, Pereira PM, Sung J, Tijani M, Wang Y, Henriques R, Collins MK, Hilgemann DW (2019). TMEM16F activation by Ca^2+^ triggers plasma membrane expansion and directs PD-1 trafficking. Scientific Reports.

[bib5] Brunner JD, Lim NK, Schenck S, Duerst A, Dutzler R (2014). X-ray structure of a calcium-activated TMEM16 lipid scramblase. Nature.

[bib6] Bushell S (2018). The structural basis of lipid scrambling and inactivation in the endoplasmic reticulum scramblase TMEM16K. bioRxiv.

[bib7] Campeau E, Ruhl VE, Rodier F, Smith CL, Rahmberg BL, Fuss JO, Campisi J, Yaswen P, Cooper PK, Kaufman PD (2009). A versatile viral system for expression and depletion of proteins in mammalian cells. PLOS ONE.

[bib8] Caputo A, Caci E, Ferrera L, Pedemonte N, Barsanti C, Sondo E, Pfeffer U, Ravazzolo R, Zegarra-Moran O, Galietta LJ (2008). TMEM16A, a membrane protein associated with calcium-dependent chloride channel activity. Science.

[bib9] Dang S, Feng S, Tien J, Peters CJ, Bulkley D, Lolicato M, Zhao J, Zuberbühler K, Ye W, Qi L, Chen T, Craik CS, Jan YN, Minor DL, Cheng Y, Jan LY (2017). Cryo-EM structures of the TMEM16A calcium-activated chloride channel. Nature.

[bib10] De Jesús-Pérez JJ, Cruz-Rangel S, Espino-Saldaña Ángeles E., Martínez-Torres A, Qu Z, Hartzell HC, Corral-Fernandez NE, Pérez-Cornejo P, Arreola J (2018). Phosphatidylinositol 4,5-bisphosphate, cholesterol, and fatty acids modulate the calcium-activated chloride channel TMEM16A (ANO1). Biochimica Et Biophysica Acta (BBA) - Molecular and Cell Biology of Lipids.

[bib11] Delling M, DeCaen PG, Doerner JF, Febvay S, Clapham DE (2013). Primary cilia are specialized calcium signalling organelles. Nature.

[bib12] Falzone ME, Malvezzi M, Lee B-C, Accardi A (2018). Known structures and unknown mechanisms of TMEM16 scramblases and channels. The Journal of General Physiology.

[bib13] Falzone ME, Rheinberger J, Lee BC, Peyear T, Sasset L, Raczkowski AM, Eng ET, Di Lorenzo A, Andersen OS, Nimigean CM, Accardi A (2019). Structural basis of Ca^2+^-dependent activation and lipid transport by a TMEM16 scramblase. eLife.

[bib14] Feng S, Dang S, Han TW, Ye W, Jin P, Cheng T, Li J, Jan YN, Jan LY, Cheng Y (2019). Cryo-EM studies of TMEM16F Calcium-Activated ion channel suggest features important for lipid scrambling. Cell Reports.

[bib15] Forschbach V, Goppelt-Struebe M, Kunzelmann K, Schreiber R, Piedagnel R, Kraus A, Eckardt KU, Buchholz B (2015). Anoctamin 6 is localized in the primary cilium of renal tubular cells and is involved in apoptosis-dependent cyst lumen formation. Cell Death & Disease.

[bib16] Grubb S, Poulsen KA, Juul CA, Kyed T, Klausen TK, Larsen EH, Hoffmann EK (2013). TMEM16F (Anoctamin 6), an anion channel of delayed Ca^2+^ activation. The Journal of General Physiology.

[bib17] Gyobu S, Ishihara K, Suzuki J, Segawa K, Nagata S (2017). Characterization of the scrambling domain of the TMEM16 family. PNAS.

[bib18] Han TW, Ye W, Bethel NP, Zubia M, Kim A, Li KH, Burlingame AL, Grabe M, Jan YN, Jan LY (2019). Chemically induced vesiculation as a platform for studying TMEM16F activity. PNAS.

[bib19] Hu Y, Kim JH, He K, Wan Q, Kim J, Flach M, Kirchhausen T, Vortkamp A, Winau F (2016). Scramblase TMEM16F terminates T cell receptor signaling to restrict T cell exhaustion. The Journal of Experimental Medicine.

[bib20] Jeng G, Aggarwal M, Yu WP, Chen TY (2016). Independent activation of distinct pores in dimeric TMEM16A channels. The Journal of General Physiology.

[bib21] Jiang T, Yu K, Hartzell HC, Tajkhorshid E (2017). Lipids and ions traverse the membrane by the same physical pathway in the nhTMEM16 scramblase. eLife.

[bib22] Kalienkova V, Clerico Mosina V, Bryner L, Oostergetel GT, Dutzler R, Paulino C (2019). Stepwise activation mechanism of the scramblase nhTMEM16 revealed by cryo-EM. eLife.

[bib23] Keramidas A, Moorhouse AJ, Schofield PR, Barry PH (2004). Ligand-gated ion channels: mechanisms underlying ion selectivity. Progress in Biophysics and Molecular Biology.

[bib24] Lam AK, Dutzler R (2018). Calcium-dependent electrostatic control of anion access to the pore of the calcium-activated chloride channel TMEM16A. eLife.

[bib25] Le T, Jia Z, Le SC, Zhang Y, Chen J, Yang H (2019a). An inner activation gate controls TMEM16F phospholipid scrambling. Nature Communications.

[bib26] Le T, Le SC, Yang H (2019b). *Drosophila* subdued is a moonlighting transmembrane protein 16 (TMEM16) that transports ions and phospholipids. Journal of Biological Chemistry.

[bib27] Lee BC, Menon AK, Accardi A (2016). The nhTMEM16 scramblase is also a nonselective ion channel. Biophysical Journal.

[bib28] Li M, Toombes GE, Silberberg SD, Swartz KJ (2015). Physical basis of apparent pore dilation of ATP-activated P2X receptor channels. Nature Neuroscience.

[bib29] Lim NK, Lam AK, Dutzler R (2016). Independent activation of ion conduction pores in the double-barreled calcium-activated chloride channel TMEM16A. The Journal of General Physiology.

[bib30] Malvezzi M, Chalat M, Janjusevic R, Picollo A, Terashima H, Menon AK, Accardi A (2013). ^Ca2+^-dependent phospholipid scrambling by a reconstituted TMEM16 ion channel. Nature Communications.

[bib31] Nguyen DM, Chen LS, Yu W-P, Chen T-Y (2019). Comparison of ion transport determinants between a TMEM16 chloride channel and phospholipid scramblase. The Journal of General Physiology.

[bib32] Paulino C, Kalienkova V, Lam AKM, Neldner Y, Dutzler R (2017a). Activation mechanism of the calcium-activated chloride channel TMEM16A revealed by cryo-EM. Nature.

[bib33] Paulino C, Neldner Y, Lam AK, Kalienkova V, Brunner JD, Schenck S, Dutzler R (2017b). Structural basis for anion conduction in the calcium-activated chloride channel TMEM16A. eLife.

[bib34] Pedemonte N, Galietta LJ (2014). Structure and function of TMEM16 proteins (anoctamins). Physiological Reviews.

[bib35] Pelz T, Drose DR, Fleck D, Henkel B, Ackels T, Spehr M, Neuhaus EM (2018). An ancestral TMEM16 homolog from *Dictyostelium discoideum* forms a scramblase. PLOS ONE.

[bib36] Peters CJ, Gilchrist JM, Tien J, Bethel NP, Qi L, Chen T, Wang L, Jan YN, Grabe M, Jan LY (2018). The sixth transmembrane segment is a major gating component of the TMEM16A Calcium-Activated chloride channel. Neuron.

[bib37] Schroeder BC, Cheng T, Jan YN, Jan LY (2008). Expression cloning of TMEM16A as a calcium-activated chloride channel subunit. Cell.

[bib38] Scudieri P, Caci E, Venturini A, Sondo E, Pianigiani G, Marchetti C, Ravazzolo R, Pagani F, Galietta LJ (2015). Ion channel and lipid scramblase activity associated with expression of TMEM16F/ANO6 isoforms. The Journal of Physiology.

[bib39] Shimizu T, Iehara T, Sato K, Fujii T, Sakai H, Okada Y (2013). TMEM16F is a component of a Ca2+-activated cl- channel but not a volume-sensitive outwardly rectifying cl- channel. American Journal of Physiology. Cell Physiology.

[bib40] Stephan AB, Shum EY, Hirsh S, Cygnar KD, Reisert J, Zhao H (2009). ANO2 is the cilial calcium-activated chloride channel that may mediate olfactory amplification. PNAS.

[bib41] Sundelacruz S, Levin M, Kaplan DL (2009). Role of membrane potential in the regulation of cell proliferation and differentiation. Stem Cell Reviews and Reports.

[bib42] Suzuki J, Umeda M, Sims PJ, Nagata S (2010). Calcium-dependent phospholipid scrambling by TMEM16F. Nature.

[bib43] Suzuki J, Fujii T, Imao T, Ishihara K, Kuba H, Nagata S (2013). Calcium-dependent phospholipid scramblase activity of TMEM16 protein family members. Journal of Biological Chemistry.

[bib44] Tian Y, Schreiber R, Kunzelmann K (2012). Anoctamins are a family of Ca2+-activated cl- channels. Journal of Cell Science.

[bib45] Watanabe R, Sakuragi T, Noji H, Nagata S (2018). Single-molecule analysis of phospholipid scrambling by TMEM16F. PNAS.

[bib46] Whitlock JM, Yu K, Cui YY, Hartzell HC (2018). Anoctamin 5/TMEM16E facilitates muscle precursor cell fusion. The Journal of General Physiology.

[bib47] Whitlock JM, Hartzell HC (2017). Anoctamins/TMEM16 proteins: chloride channels flirting with lipids and extracellular vesicles. Annual Review of Physiology.

[bib48] Xiao Q, Yu K, Perez-Cornejo P, Cui Y, Arreola J, Hartzell HC (2011). Voltage- and calcium-dependent gating of TMEM16A/Ano1 chloride channels are physically coupled by the first intracellular loop. PNAS.

[bib49] Yang YD, Cho H, Koo JY, Tak MH, Cho Y, Shim WS, Park SP, Lee J, Lee B, Kim BM, Raouf R, Shin YK, Oh U (2008). TMEM16A confers receptor-activated calcium-dependent chloride conductance. Nature.

[bib50] Yang H, Kim A, David T, Palmer D, Jin T, Tien J, Huang F, Cheng T, Coughlin SR, Jan YN, Jan LY (2012). TMEM16F forms a Ca^2+^-activated cation channel required for lipid scrambling in platelets during blood coagulation. Cell.

[bib51] Ye W, Han TW, Nassar LM, Zubia M, Jan YN, Jan LY (2018). Phosphatidylinositol-(4, 5)-bisphosphate regulates calcium gating of small-conductance cation channel TMEM16F. PNAS.

[bib52] Yifrach O (2004). Hill coefficient for estimating the magnitude of cooperativity in gating transitions of voltage-dependent ion channels. Biophysical Journal.

[bib53] Yu Y, Kuan AS, Chen TY (2014). Calcium-calmodulin does not alter the anion permeability of the mouse TMEM16A calcium-activated chloride channel. The Journal of General Physiology.

[bib54] Yu K, Whitlock JM, Lee K, Ortlund EA, Cui YY, Hartzell HC (2015). Identification of a lipid scrambling domain in ANO6/TMEM16F. eLife.

[bib55] Zaitseva E, Zaitsev E, Melikov K, Arakelyan A, Marin M, Villasmil R, Margolis LB, Melikyan GB, Chernomordik LV (2017). Fusion stage of HIV-1 entry depends on Virus-Induced cell surface exposure of phosphatidylserine. Cell Host & Microbe.

