# Supplementary material for: Dynamic change of electrostatic field in TMEM16F permeation pathway shifts its ion selectivity
Source: eLife. 2019 Jul 18;8:e45187. doi: 10.7554/eLife.45187 (PMC6690719; doi:10.7554/eLife.45187)
Supplement: Transparent reporting form [file elife-45187-transrepform.docx]

***eLife’s* transparent reporting form**

We encourage authors to provide detailed information *within their submission* to facilitate the interpretation and replication of experiments. Authors can upload supporting documentation to indicate the use of appropriate reporting guidelines for health-related research (see [EQUATOR Network](http://www.equator-network.org/%20)), life science research (see the [BioSharing Information Resource](https://biosharing.org/)), or the [ARRIVE guidelines](http://www.plosbiology.org/article/info:doi/10.1371/journal.pbio.1000412) for reporting work involving animal research. Where applicable, authors should refer to any relevant reporting standards documents in this form.

If you have any questions, please consult our Journal Policies and/or contact us: [editorial@elifesciences.org](mailto:editorial@elifesciences.org).

**Sample-size estimation**

- You should state whether an appropriate sample size was computed when the study was being designed
- You should state the statistical method of sample size computation and any required assumptions
- If no explicit power analysis was used, you should describe how you decided what sample (replicate) size (number) to use

Please outline where this information can be found within the submission (e.g., sections or figure legends), or explain why this information doesn’t apply to your submission:

Every experiment was replicated at least three times (N≥3), and mostly ~5 times. Most data were displayed as scatter plots (one dot, one value). Most experiments were analyzed with Student's t test or ANOVA, unless otherwise stated. All statistical methods were listed in figure legends. n.s. P>0.05, * P<0.05, **P<0.01, ***P<0.001, ****P<0.0001.

**Replicates**

- You should report how often each experiment was performed
- You should include a definition of biological versus technical replication
- The data obtained should be provided and sufficient information should be provided to indicate the number of independent biological and/or technical replicates
- If you encountered any outliers, you should describe how these were handled
- Criteria for exclusion/inclusion of data should be clearly stated
- High-throughput sequence data should be uploaded before submission, with a private link for reviewers provided (these are available from both GEO and ArrayExpress)

Please outline where this information can be found within the submission (e.g., sections or figure legends), or explain why this information doesn’t apply to your submission:

Every experiment was replicated at least three times (N≥3), and mostly ~5 times. Critical experiments were performed on separate days to ensure the consistency.

Every recording was a biological replication, since we used different batches of cells for each of them. Cells from the same dish were not repeatedly used.

We used scatter plots for most of the experiments. Each dot represents a biological replicate.

we did not encounter apparent outliers. In other words, we used all the obtained data in listed experiments.

Note: As we mentioned in the methods: "Cells were disposed of if they were not confluent in the flask in 4 days, their morphologies deviated from pictures in instructions (ThermoFisher website), or if the batch had been used for 3 months, whichever coming first, but were not in particular tested for mycoplasma contamination."

After we submitted our manuscript we were suggested adding the information of cell authentication and the test for mycoplasma contamination by Elife editorial. The above describes the “real” details of our experimental procedures. Our data are reproducible among cell batches across many months, so it is unlikely that the cells are contaminated and meanwhile the contamination affected our results. We did not think we should thaw new cells from liquid nitrogen to perform these experiments since the preserved cells do not represent the cells being used in the experiments in the manuscript, in any case if the cells being used were "really" contaminated.

**Statistical reporting**

- Statistical analysis methods should be described and justified
- Raw data should be presented in figures whenever informative to do so (typically when N per group is less than 10)
- For each experiment, you should identify the statistical tests used, exact values of N, definitions of center, methods of multiple test correction, and dispersion and precision measures (e.g., mean, median, SD, SEM, confidence intervals; and, for the major substantive results, a measure of effect size (e.g., Pearson's r, Cohen's d)
- Report exact p-values wherever possible alongside the summary statistics and 95% confidence intervals. These should be reported for all key questions and not only when the p-value is less than 0.05.

Please outline where this information can be found within the submission (e.g., sections or figure legends), or explain why this information doesn’t apply to your submission:

Most experiments were analyzed with Student's t test or ANOVA, unless otherwise stated. All statistical methods were listed in figure legends. n.s. P>0.05, * P<0.05, **P<0.01, ***P<0.001, ****P<0.0001.

We used scatter plots for most of the experiments. Each dot represents a biological replicate.

Data were presented as mean +/- SEM, as mentioned in the Methods.

For most experiments the P is less than 0.01 or 0.001. If *P* is between 0.01 and 0.05, we can list it upon reviewers' requirements.

(For large datasets, or papers with a very large number of statistical tests, you may upload a single table file with tests, Ns, etc., with reference to sections in the manuscript.)

**Group allocation**

- Indicate how samples were allocated into experimental groups (in the case of clinical studies, please specify allocation to treatment method); if randomization was used, please also state if restricted randomization was applied
- Indicate if masking was used during group allocation, data collection and/or data analysis

Please outline where this information can be found within the submission (e.g., sections or figure legends), or explain why this information doesn’t apply to your submission:

Each recording was performed on a cell in a different dish. For critical experiments, recordings were performed on different days.

**Additional data files (“source data”)**

- We encourage you to upload relevant additional data files, such as numerical data that are represented as a graph in a figure, or as a summary table
- Where provided, these should be in the most useful format, and they can be uploaded as “Source data” files linked to a main figure or table
- Include model definition files including the full list of parameters used
- Include code used for data analysis (e.g., R, MatLab)
- Avoid stating that data files are “available upon request”

Please indicate the figures or tables for which source data files have been provided:

We use scatter plots for most of the experiments, which already represent "original data".

All the data analyses were performed with Graphpah and/or Origin, which were mentioned in Methods.
